# Askival: An altered feldspathic cumulate sample in Gale crater

**DOI:** 10.1111/maps.13933

**Published:** 2022-12-04

**Authors:** Donald Lewis Bowden, John C. Bridges, Agnes Cousin, William Rapin, Julia Semprich, Olivier Gasnault, Olivier Forni, Patrick Gasda, Debarati Das, Valerie Payré, Violaine Sautter, Candice C. Bedford, Roger C. Wiens, Patrick Pinet, Jens Frydenvang

**Affiliations:** ^1^ School of Physics and Astronomy University of Leicester LE1 7RH Leicester UK; ^2^ Institut de Recherche en Astrophysique et Planétologie Université de Toulouse CNRS, CNES 31400 Toulouse France; ^3^ AstrobiologyOU, School of Environment, Earth and Ecosystem Sciences The Open University MK7 6AA Milton Keynes Walton Hall UK; ^4^ Los Alamos National Laboratory 87545 New Mexico Los Alamos USA; ^5^ Department of Earth and Planetary Sciences McGill University H3A 0E8 Quebec Montreal Canada; ^6^ Department of Earth and Environmental Sciences The University of Iowa 52242 Iowa Iowa City USA; ^7^ Muséum national d'Histoire naturelle 75005 Paris France; ^8^ Lunar and Planetary Institute USRA 77058 Texas Houston USA; ^9^ Astromaterials Research and Exploration Science NASA Johnson Space Center 77058 Texas Houston USA; ^10^ University of Copenhagen DK‐1165 Copenhagen Denmark

## Abstract

Askival is a light‐toned, coarsely crystalline float rock, which was identified near the base of Vera Rubin Ridge in Gale crater. We have studied Askival, principally with the ChemCam instrument but also using APXS compositional data and MAHLI images. Askival and an earlier identified sample, Bindi, represent two rare examples of feldspathic cumulate float rocks in Gale crater with >65% relict plagioclase. Bindi appears unaltered whereas Askival shows textural and compositional signatures of silicification, along with alkali remobilization and hydration. Askival likely experienced multiple stages of alteration, occurring first through acidic hydrolysis of metal cations, followed by deposition of silica and possible phyllosilicates at low T and neutral‐alkaline pH. Through laser‐induced breakdown spectroscopy compositional analyses and normative calculations, we suggest that an assemblage of Fe‐Mg silicates including amphibole and pyroxene, Fe phases, and possibly Mg‐rich phyllosilicate are present. Thermodynamic modeling of the more pristine Bindi composition predicts that amphibole and feldspar are stable within an upper crustal setting. This is consistent with the presence of amphibole in the parent igneous rocks of Askival and suggests that the paucity of amphiboles in other known Martian samples reflects the lack of representative samples of the Martian crust rather than their absence on Mars.

## Introduction

A series of igneous float rocks have been identified in Gale crater that consist predominantly of basalts and trachybasalts (Cousin et al., 2017; Edwards et al., 2017) but with other examples of evolved bulk rock compositions having total alkali contents up to 14 wt% (Sautter et al., 2015; Schmidt et al., 2013). These samples are likely the result of fractionation of basaltic magma and are reminiscent of intraplate magmatism on Earth (Edwards et al., 2017; Udry et al., 2018).

**Fig. 1:**
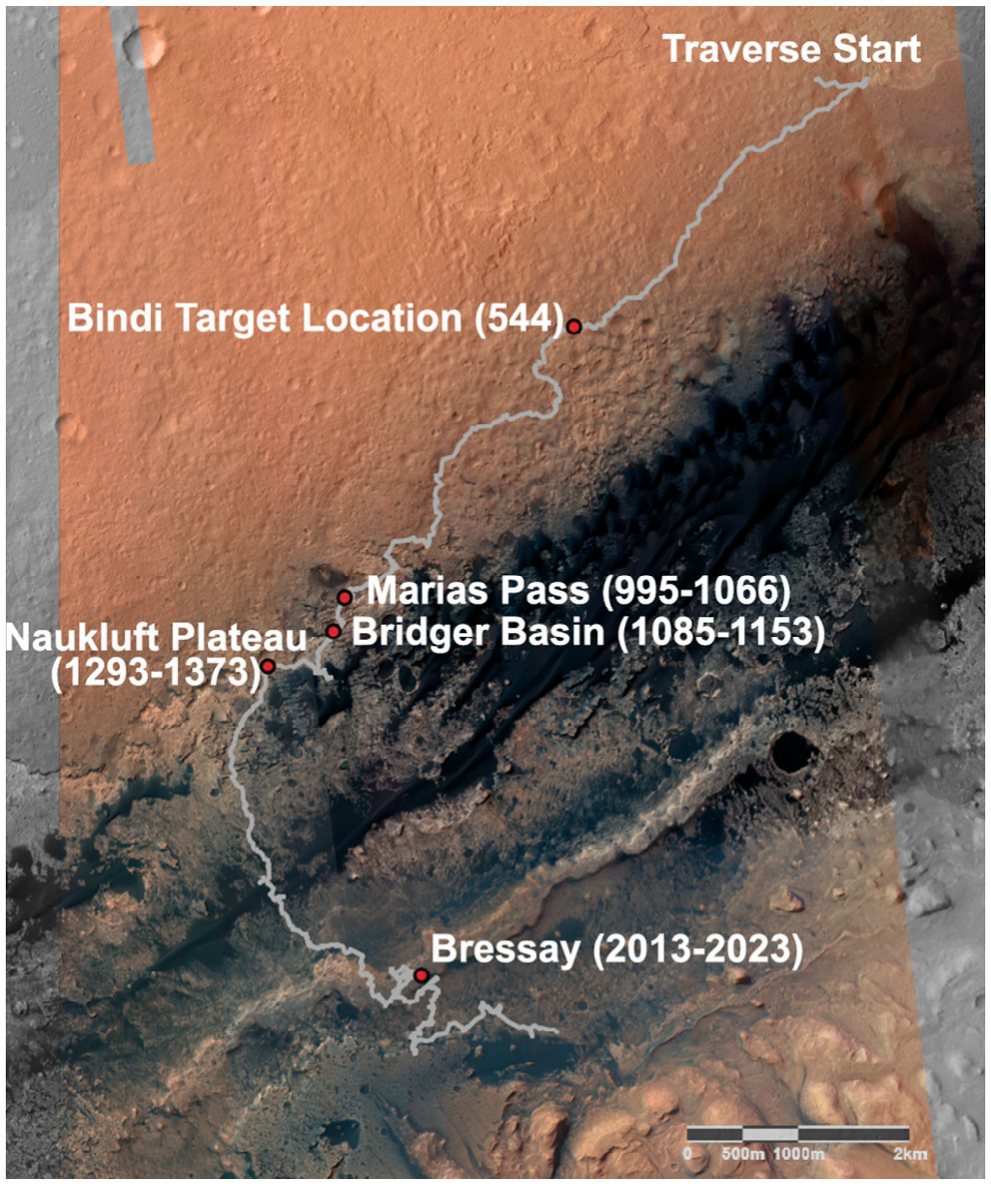
Traverse map showing the Bressay site where Askival is located, as well as the location of Bindi. In addition, the Marias Pass, Bridger Basin, and Naukluft Plateau locales, at which evidence of diagenetic silica enrichment was encountered (Frydenvang et al., 2017), are marked. Parentheses indicate mission sols covering each area. (Color figure can be viewed at wileyonlinelibrary.com.)

**Fig. 2:**
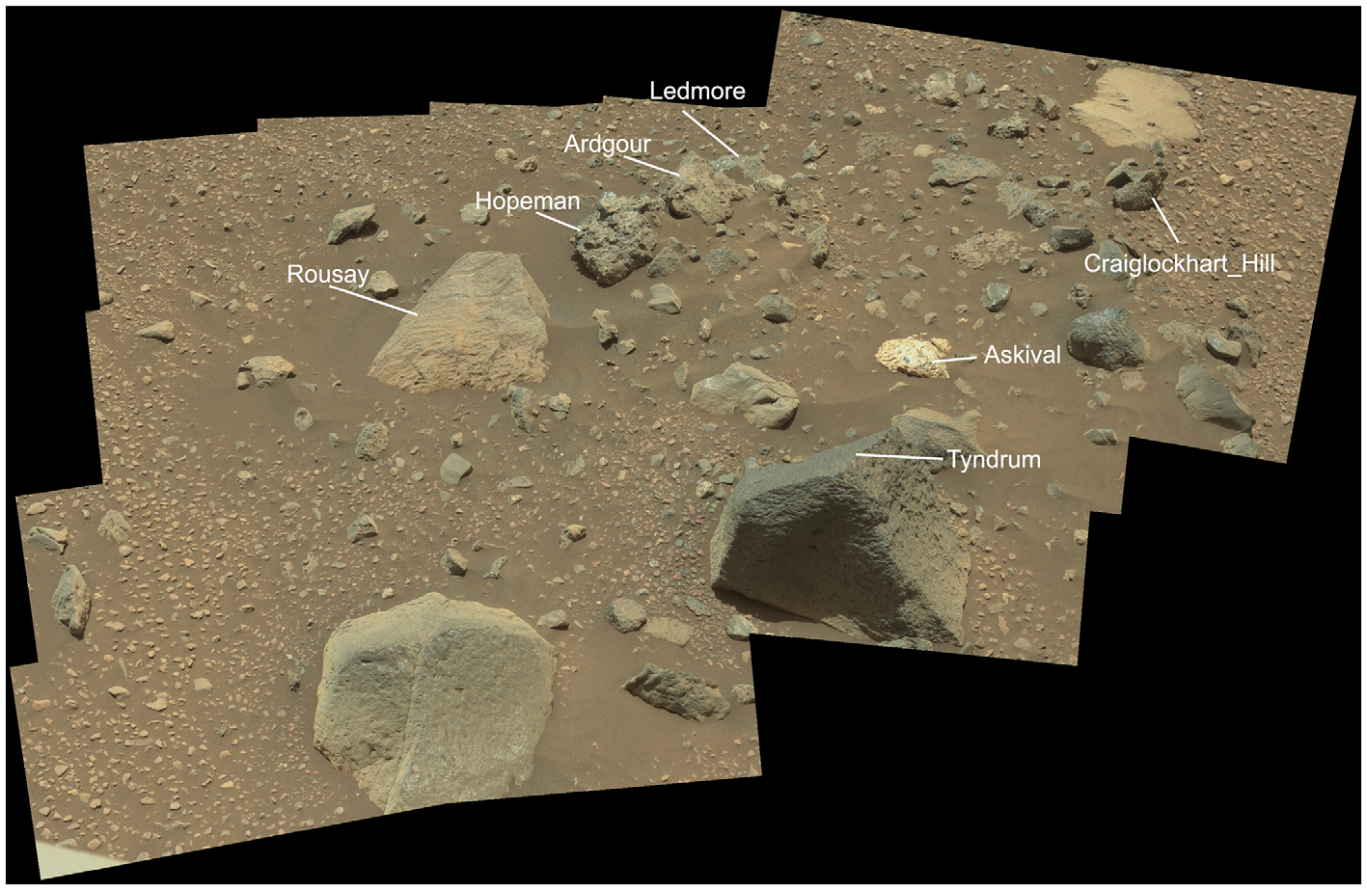
MastCam mosaic CX02016MR0691552 of Bressay workspace with labeled scientific targets. The Askival sample is ~20 cm in length. Source images: NASA/JPL‐Caltech/MSSS, DOI 10.17189/1520328. Context mosaic produced by Mars Analyst's Notebook. (Color figure can be viewed at wileyonlinelibrary.com.)

However, one sample—named Askival—is anomalous and represents a light‐toned, coarsely crystalline float rock which was identified on sol 2018, at the Bressay boulder field locality, near the base of Vera Rubin Ridge (Figs. 1 and 2). The Curiosity rover was able to obtain MastCam, Mars Hand Lens Imager (MAHLI), and ChemCam Remote Micro‐Imager (RMI) imagery at high resolution and color, together with ChemCam laser‐induced breakdown spectroscopy (LIBS) and Alpha Particle X‐ray Spectrometer (APXS) compositional data on Askival. Data show an extremely high silica‐rich composition that is unlikely to be the result of purely igneous processes.

We describe the Askival sample using the rover data with the aim of distinguishing between alteration overprints and its original mineral assemblage and compositional signatures. To do this, we compare to previously described samples interpreted as pristine feldspar‐rich igneous rocks, in particular the Bindi sample from sol 544 (Cousin et al., 2017; Edwards et al., 2017) (location shown on Fig. 1). Bindi is believed to be a feldspathic cumulate related to the basalt–trachybasalt igneous rocks in Gale. A key part of the investigation of Askival is to reconstruct alteration processes that significantly modified its original mineralogy. We use ChemCam laser spots to analyze the sub‐mm scale mineral intergrowths within the rock. We attempt to constrain the primary and secondary mineral assemblages in Askival through comparison of LIBS data with candidate mineral and normative compositions. On the basis of the compositional and textural data informing phase equilibria modeling, we also consider the stability of different mineral assemblages for the mafic phases in Askival. We also discuss the processes that altered Askival. This is an important issue as Askival is the first feldspathic rock found on Mars with such evidence for alteration. Based on the discovery of its possible unaltered counterparts, it has been proposed that feldspar‐rich rocks could be a large fraction of the crust in the ancient highlands (Sautter et al., 2015). Feldspathic rocks have been challenging to identify from orbit, due to their paucity of spectral signatures. Their alteration products have been predicted from orbit in specific localities (Ehlmann & Edwards, 2014), but there has been no documentation of partial alteration on this type of rock on the surface of Mars. Askival provides a first view of an alteration history for Martian feldspathic rocks.

## Methods

### ChemCam and LIBS

The Mars Science Laboratory (MSL) ChemCam is a laser‐LIBS instrument with accompanying RMI for target characterization through photographic imaging. ChemCam is the first LIBS instrument employed on a space mission. The motivation for choosing this type of instrument on the MSL mission was the need for a remote sensing instrument that could provide mineralogical data through rock chemistry, improving on previous rover missions, which had carried multispectral imaging cameras and infrared spectrometers for remote sensing and relied on contact instruments for detailed chemical or mineralogical analysis. The ability to gather this type of data through remote sensing carries large benefits to science operations through both faster data collection and expansion of the range of targets that are available as targets for investigation (Maurice et al., 2012; Wiens et al., 2012).

The fundamental principle of LIBS is the use of a laser pulse to create a small region of energetically excited plasma on the surface of a target. As the atoms return to the ground state, characteristic photons are emitted which are observed using three spectrometers across ultraviolet, violet, and visible/near‐infrared (UV‐VIO‐NIR) and analyzed to determine the chemical content of the target. In order to create an LIBS plasma on a typical Martian target, the required power density was determined to be >1 GW m^−2^ (Maurice et al., 2012), which the ChemCam laser unit is capable of delivering at distances up to several meters, although most analyses are done from the optimal distance of ~3 m (including Askival which was performed at 2.5–2.7 m, and Bindi which was performed at 2.74 m). The laser uses a wavelength of 1067 nm, with an adjustable repetition rate set at 3 Hz.

Each target is analyzed by a raster pattern of observation points which in turn consist of usually 30 laser pulses and corresponding spectral analyses. LIBS plasma light is collected through the ChemCam telescope, which then delivers the incident photons through fiber‐optic cable into the rover body unit, which contains three crossed Czerny–Turner spectrometers, each covering a portion of the total spectral range, 240–850 nm (Wiens et al., 2012).

The repeated firing of the laser at a specific point on the incident target gradually clears the ubiquitous Martian dust coating (Lasue et al., 2018; Maurice et al., 2016); typically the first five pulses are sufficient to fully remove the dust covering from the target surface (Wiens et al., 2012). These first five pulses are then excluded from the compositional analysis of the observation point. Observation point analyses are averages of the spectra conducted at a given raster point. Raster geometries are typically planned in linear (5 by 1, 10 by 1, and less often 3 by 1 or 3 by 3) configurations. Point footprints range from 350 to 750 μm in size depending on distance from the rover (Maurice et al., 2016; Wiens et al., 2012). In this paper, #1 refers to the first point number in a LIBS raster, #2 to the second point, etc.

### Alpha Particle X‐Ray Spectrometer

The The APXS instrument is a contact science instrument mounted on the end of the Curiosity rover's robotic arm. The APXS uses a radioactive ^244^Cm source to induce X‐ray emission in material contacted by the instrument. This instrument is an improved version of those carried by the Mars Exploration Rovers and Pathfinder lander (Campbell et al., 2012; Gellert & Clark, 2015; Gellert et al., 2006). Subjected to the alpha particle and X‐ray flux emitted by the radioactive source, the constituent atoms of a contacted target emit characteristic X‐rays, which are detected by the instrument's X‐ray detector, recording their energies. The APXS typically measures a sample area 1.7 cm across, producing an averaged spectrum from all mineral grains in its field of view (Gellert & Clark, 2015).

Due to the dust covering present across the Martian surface, the APXS is often used in tandem with the Dust Removal Tool (DRT) brush to remove the dust (Berger, Gellert, et al., 2020; Berger, Schmidt, et al., 2020). However, DRT brushing is not possible on small float rocks like Askival and so we will consider the results for the potential to have dust contamination. APXS analyzed Askival with two measurements with durations of 12 and 15 min and one overnight measurement with a duration of 5 h 30 min on sols 2018–2019. Bindi was not analyzed by APXS.

### Mars Hand Lens Imager and ChemCam RMI Imager

The MAHLI is an optical imaging instrument mounted on the Curiosity rover's robotic arm. MAHLI's capabilities as a high‐resolution imager were developed with the goal of providing images of targets with sufficient detail to allow textural features such as grain size, shape, and structural features to be observed. White and UV light are provided by light‐emitting diodes in tandem with the imager, providing illumination and observation of target luster in visible light as well as potential observations of fluorescence under UV light (Edgett et al., 2012). MAHLI images are produced using a focus merging process and have a field of view of 1200 × 1600 pixels. At the closest operating distance of 2.1 cm, this corresponds to a pixel scale of 14.3 μm, providing enough detail to image submillimeter scale textural features and provide accurate depictions of grain shape and size.

The ChemCam analysis sequence also collects RMI images with a submillimeter resolution (~90 μm at the target distances for Askival) before and after LIBS analyses (Le Mouélic et al., 2015; Maurice et al., 2012). Some of the RMI images we use in this paper are colorized, using Mastcam images for the color (Le Mouélic et al., 2015).

MAHLI and RMI images were annotated manually with the assistance of edge detection tools in commercially available image editing software (Adobe Photoshop) in order to categorize and quantify the visible area of different mineral phases.

### Data Analysis and Calibration of ChemCam Data

Interpretation of ChemCam data is a multistage process. An initial preprocessing stage is required in order to subtract ambient light from the image, remove noise and the electron continuum signal, as well as to apply corrections to the data based on the wavelength, distance to target, and instrument response. Following preprocessing, multivariate analysis techniques are used to derive a set of chemical abundances from the received spectra. Historically, partial least squares (PLS) algorithms have been used to analyze this information, starting with a PLS2 algorithm used in initial calibration and early rover operations (Clegg et al., 2009; Wiens et al., 2013). More recently, this has developed into a “sub‐model” PLS technique (Anderson et al., 2017), which is used in conjunction with independent component analysis (ICA; Forni et al., 2013) in order to provide a more accurate compositional analysis across the major oxides (Clegg et al., 2017).

In order to support these analysis techniques, initial calibration of the ChemCam instrument was performed on a set of 69 geochemical standard targets to create a spectral library (Wiens et al., 2013). This library was updated later into the mission in order to better reflect the types of targets found in Gale crater, expanding to 408 standard targets (Clegg et al., 2017). We use the LIBS analytical accuracy and precision as described in Bedford et al. (2019). Accuracy is determined by the root‐mean‐square error product of prediction for representative geological samples that share abundances similar to those in the calibration data set (Clegg et al., 2017). ChemCam instrument precision is calculated as the variation observed across the shots that make up the average spectrum for each observation point (Blaney et al., 2014; Mangold et al., 2015). These errors are displayed as error bars on the figures. The optimum distance for accurate LIBS analyses across the range of major elements is about 3 m from target to LIBS telescope (Maurice et al., 2016; Wiens et al., 2021).

In some cases, the unnormalized sum of oxide weights is significantly below or above 100%. This can be the result of statistical variation in the multivariate routine as well as target compositions which differ significantly to the reference compositions used in the analysis. Spectral variation due to distance, matrix effects, and laser coupling can further compound this effect and cases where the modeled sum of oxides is significantly below 100% are typically representative of targets with chemical components which are not accounted for by the standard analysis routines (Maurice et al., 2016); of particular relevance to the Askival sample are SO_3_ and OH anions.

#### LIBS Hydrogen Analyses

The hydrogen signal was obtained from the spectra by fitting the Balmer alpha emission peak at 656.6 nm. The processing used for Martian spectra has been described first in Schröder et al. (2015), then revised with laboratory experiments in Rapin, Meslin, et al. (2017), and summarized here. It includes a multi‐Lorentz fitting method to extract the hydrogen peak area from the interference with a nearby carbon peak and a linear baseline removal. The area of the carbon peak (C I at 247.9 nm) related to the breakdown of the atmospheric CO_2_ and the area of the oxygen triplet forming a single peak (O I at 778.5, 778.6, and 778.8 nm) also partly related to the atmosphere are used for the normalization of the hydrogen signal. Signal normalization, which here consists of dividing the hydrogen peak area by the carbon peak area, is important to compensate for undesired variations of the LIBS signal due to different parameters (for instance, laser‐target coupling, laser focus, and distance to target). Normalization to both the carbon and oxygen peaks was proven to be a satisfactory normalization proxy for the hydrogen signal, even though precautions must be taken when comparing different measurement conditions (Schröder et al., 2019). Finally, a correction factor is applied to the normalized signal in order to account for the difference of instrument response between the laboratory and flight model instruments (Rapin, Meslin, et al., 2017).

Rapin, Bousquet, et al. (2017) have shown that the hydrogen LIBS signal can be affected by surface texture roughness effects. The hydrogen signal is enhanced by a factor that varies depending on laser surface geometry at the LIBS target point when the roughness scale approaches the size of the laser beam, notably near the millimeter scale. This effect is specific to the hydrogen signal and does not affect the emission of other elements (Rapin, Bousquet, et al., 2017). Nevertheless, in spite of the complications of roughness, a consistently observed increase of hydrogen signal cannot be attributed solely to the effect of roughness. Indeed, the roughness effect does not only yield high hydrogen signal points, it also produces points without an enhanced hydrogen signal. Therefore, if a group of points on a specific target material all present elevated hydrogen, it likely represents real enhanced hydration in the target material.

#### Hierarchical Clustering Analysis

In addition to the comparison of the abundances by pair of elements, and to take into account the multivariate dimension of the chemical measurements, a clustering analysis was conducted on the 23 ChemCam analyses from Askival. The calculation is based on the ICA products, one of the two components going into the calculation of ChemCam abundances as explained above, using the DIANA divisive hierarchical clustering method (Kaufman & Rousseeuw, 2009). This is implemented in the Cluster package (Maechler et al., 2022) for the statistical software R 3.5.2 (R Core Team, 2018). Dissimilarity between clusters was determined using Euclidian distance. The main output is a dendrogram tree showing how the data points can be best split in two groups, then each group in two subgroups, and repetitively until all the points are separated. The comparison of each step with the major oxide compositions indicates which chemical elements drive the division in subgroups.

#### Normative Calculations

In order to assess the possible mineralogical assemblage of Askival, we have used a normative calculation, starting from the composition of each data point of interest. These calculations are a method of predicting the idealized set of minerals that constitute material in the LIBS point. This methodology is usually applied to largely anhydrous, igneous rocks, via the CIPW technique, which gives molar mineral estimates.

Here, we have used an approach based on the same computational sequence as the CIPW norm (Kelsey, 1965), but have included likely hydrated phases such as ferrohastingsite amphibole (hornblende) and muscovite, and have excluded certain minerals that would otherwise compete with these additional minerals for chemical assignment, for example, orthoclase was replaced in the sequence by muscovite in some calculations, in order to better simulate the Askival composition. We also included the compositions of two known examples of Mg‐rich phyllosilicates from the Lafayette Martian meteorite (Hicks et al., 2014). Because our analysis deviated from standard methodology, multiple alternate normative calculations were performed for each LIBS point composition, so that the viability of different assemblages could be assessed.

As an indicator of the quality of calculations, residuals were calculated for each assemblage, with the goal of keeping residual chemistry below 2 wt%. The normative calculations attempt to assign all available chemistry to crystalline mineral phases, which assumes there is no amorphous phase, despite this being a common product of altered mafic minerals in Gale crater (Rampe et al., 2020). Therefore, normative mineral molar abundances are not definitive identifications, but they are a guide to which mineral phases may be present. The normative calculations are documented on a spreadsheet within the supplementary materials to this paper.

### Perple_X Modeling

On the basis of major element compositions and the normative calculations, we explore the possible presence of some amphibole content in Askival with phase equilibria modeling to predict stability conditions. Phase diagrams were calculated with the Gibbs free energy minimization software Perple_X 6.9.0 (Connolly, 2005) using the internally consistent thermodynamic data set of Holland and Powell (1998, 2011). The oxides MnO, Cr_2_O_3_, and P_2_O_5_ were not included in the calculation because of their low abundance. Halogens were not included due to their absence in available solid solution models. Sulfur was excluded since it is not quantified by the ChemCam data and all sulfur detected by APXS in Askival is interpreted to be associated with low‐temperature alteration as calcium sulfate. The bulk water content in Martian igneous samples is estimated to be low (e.g., Leshin et al., 1996; Usui et al., 2012, 2015); however, Martian magmas may have lost water due to partial degassing (e.g., McSween & Harvey, 1993) and the estimated pre‐eruptive water content ranges from dry to 2 wt% H_2_O (Giesting et al., 2015; McCubbin et al., 2010, 2012; Usui et al., 2012, 2015). As a starting composition, we used the Bindi light phase composition (Bindi #5), normalized to 100%, and with the addition of 0.5 wt% of H_2_O. Ten wt% of the total iron was assumed to be Fe_2_O_3_ (e.g., Edwards et al., 2017) which was added to Perple_X by the following existing components: 2 FeO + 0.5 O_2_. The starting composition of our calculations is hence (wt%): 50.5 SiO_2_, 0.94 TiO_2_, 13.4 Al_2_O_3_, 2.06 Fe_2_O_3_, 16.8 FeO, 1.96 MgO, 10.2 CaO, 3.38 Na_2_O, 0.99 K_2_O, 0.5 H_2_O, which was then normalized by Perple_X. Phase stability fields were calculated in the temperature range of 500–1000 °C at pressures of 1–2000 bars. The following solid solution models were used: basaltic melt, augite (clinopyroxene), and amphibole (Green et al., 2016); olivine (Holland & Powell, 1998); ilmenite (White et al., 2000); spinel (White et al., 2002); and feldspar (Holland & Powell, 2003). The amphibole model uses the formula A[M1]_3_[M2]_2_[M4]_2_[T1]_4_(OH)_2_Si_4_O_22_ allowing for mixing on the following sites: A = Na, K (and with vacancies); M1 = Mg, Fe^2+^; M2 = Mg, Fe^2+^, Fe^3+^, Al, Ti; M4 = Mg, Fe^2+^, Na, Ca; T1 = Al, Si; OH = O, OH (Green et al., 2016). The composition of the modeled amphibole will hence vary with pressure and temperature. Biotite endmembers annite, phlogopite, and eastonite were excluded from the calculation to focus on amphibole formation. Quartz and H_2_O were included as pure phases.

## Results

The Askival and Bindi samples described in this paper were investigated at two separate localities on the rover's traverse: Askival was identified at Bressay (sols 2018–2020) and Bindi on the Gale plains at the approach traverse to the Kimberley site (sol 544), as shown in Fig. 1. Williams et al. (2020) described the geomorphology of the Bressay area. They noted that Bressay is a roughly elliptical (<200 m^2^) grouping of heterolithic float rocks forming an apron of material on top of the Vera Rubin Ridge, arranged orthogonally to the long axis of the Vera Rubin Ridge (Fig. 1). The largest stones are concentrated in an area <3 m^2^, with clast dimensions 15–25 cm across. Float rock types present at Bressay are diverse, with both igneous (one quarter of float rocks) and sedimentary (three quarters of float rocks; Williams et al., 2020) (Fig. 2).

### Textures

#### Askival

**Fig. 3:**
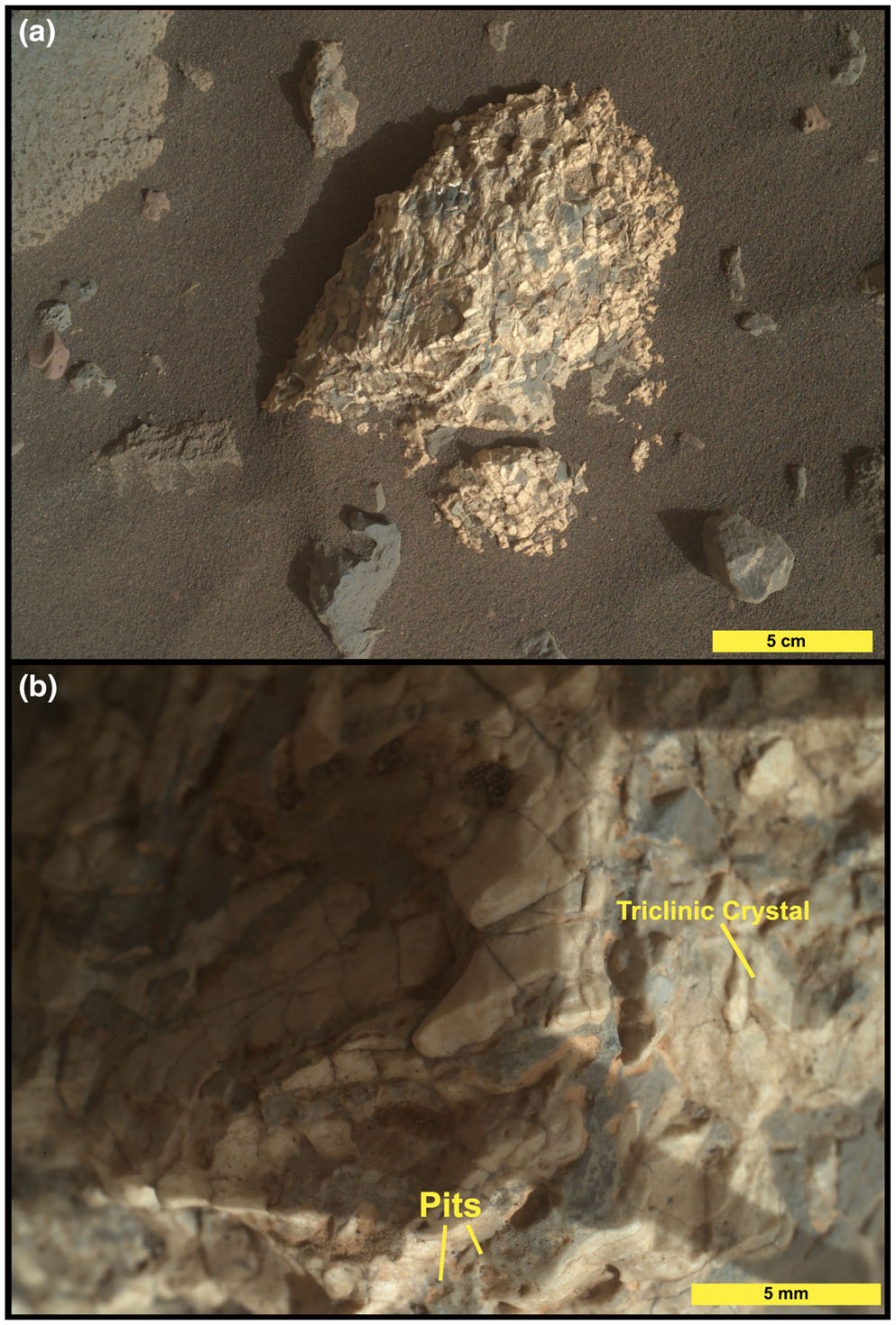
MAHLI images of Askival. a) Context image from ~25 cm standoff (sequence ID 000706). b) Close‐up image showing surface texture (sequence ID 00656). Examples of pitting in dark material and of triclinic crystal termination are labeled. (Color figure can be viewed at wileyonlinelibrary.com.)

**Fig. 4:**
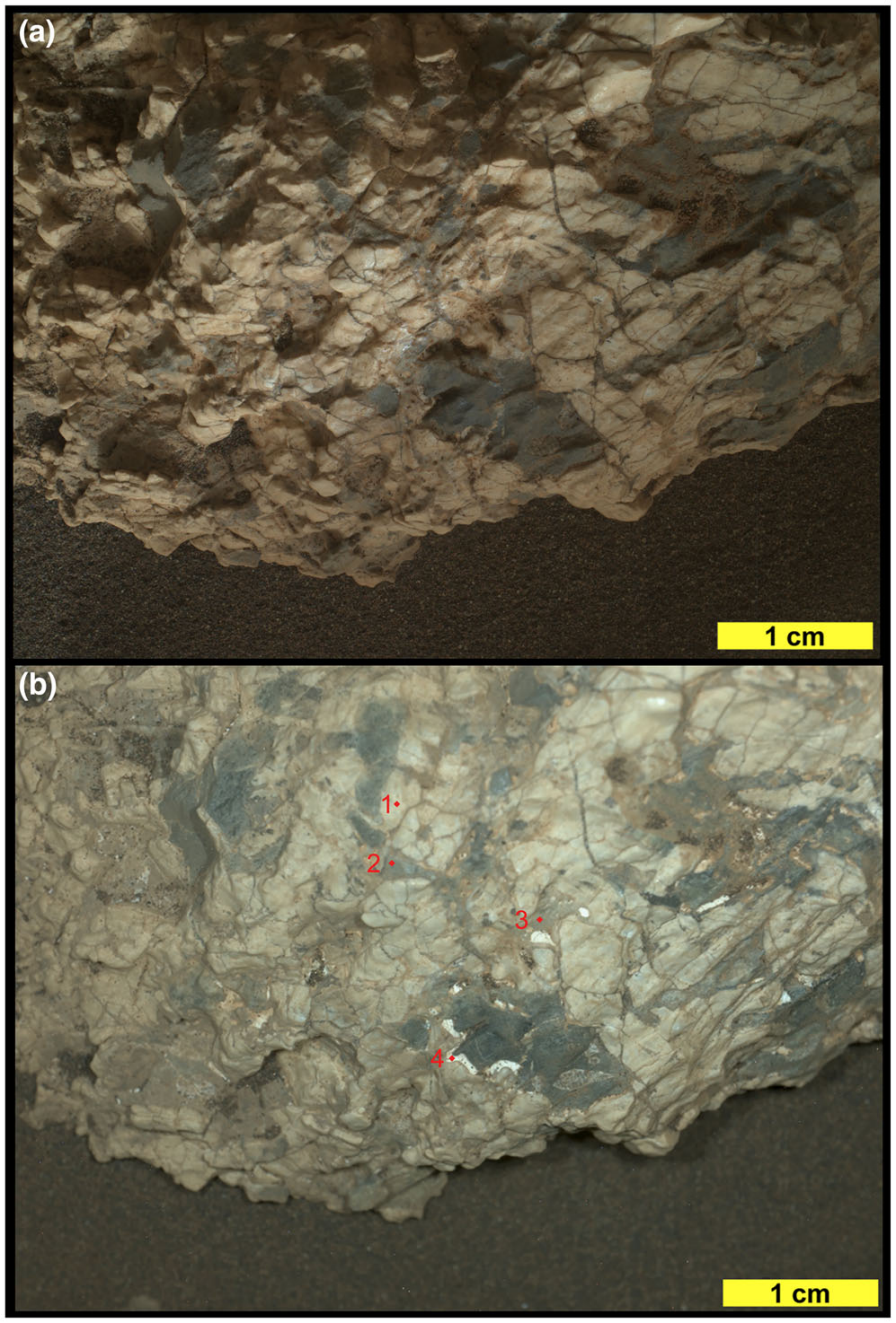
MAHLI images of Askival with differing illumination. a) Daytime illumination (sequence ID 000458). b) Nighttime with LED illumination (sequence ID 000739). Red markers indicate examples of phases 1–4 (see the section Askival and Table 1). (Color figure can be viewed at wileyonlinelibrary.com.)

Askival is a partially buried rock of approximately 10 cm visible diameter. The surface is uneven and angular, consisting of light‐ and dark‐toned phases of up to cm scale (Fig. 3). There is evidence of extensive fracturing throughout the sample, with veins in the light‐toned phase that are infilled with darker material (Figs. 3 and 4).

**Fig. 5:**
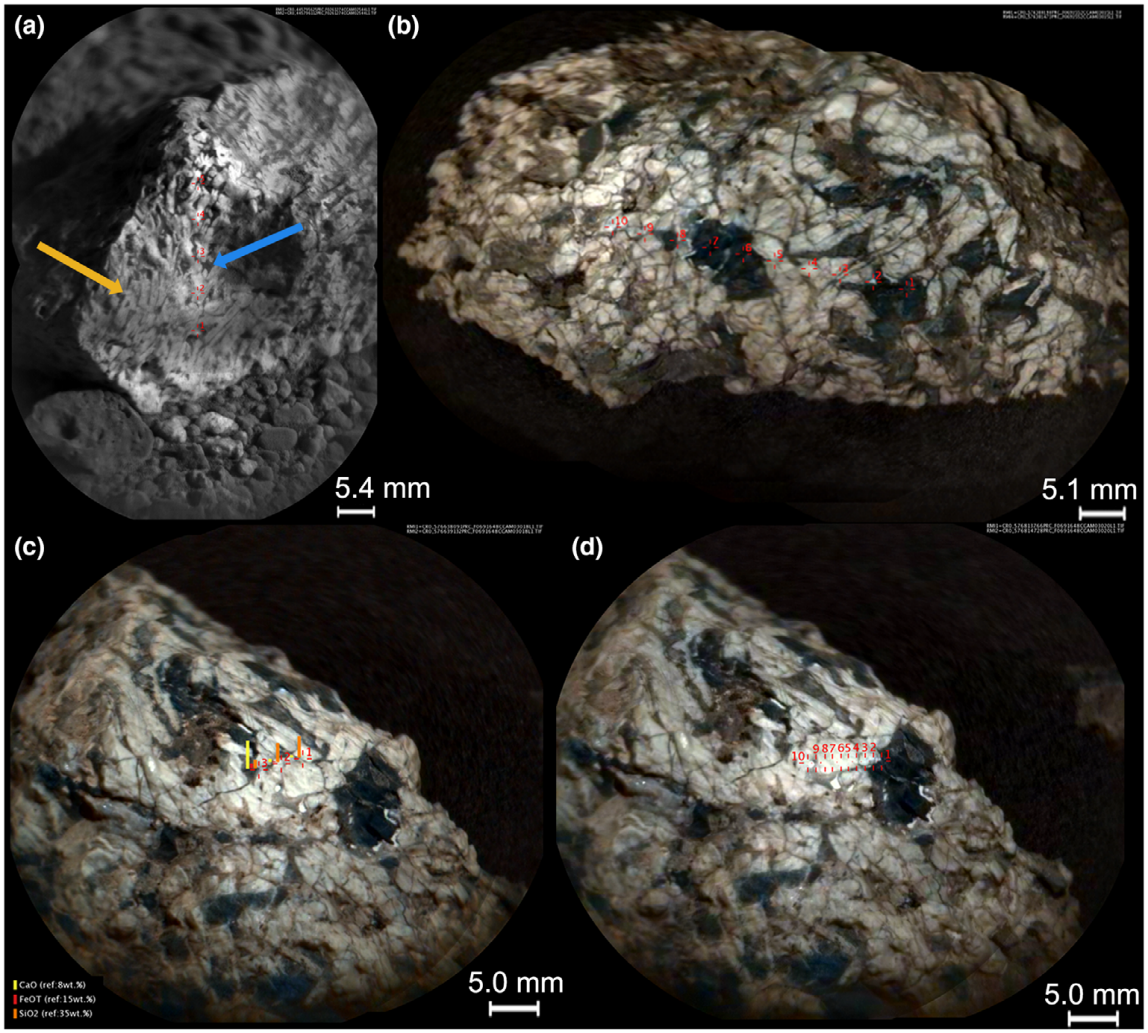
a) ChemCam RMI image of Bindi, with markers showing location of ChemCam target points. Yellow arrow indicates characteristic plagioclase lathe forms in phase 1. Blue arrow indicates darker interstitial phase 2. b–d) Colorized RMI images from Mastcam color merge (Le Mouélic et al., 2015) of Askival, with markers showing location of ChemCam target points in sequences Askival (b), Askival_2 (c), and Askival_3 (d). (Color figure can be viewed at wileyonlinelibrary.com.)

Four distinct phases have been identified based on RMI and MAHLI images (Figs. 3, 4, 5). Phase 1, the most abundant phase, is light toned and coarse grained. Exact grain size at formation is difficult to constrain due to fracturing of phase 1, but some examples of single crystals up to 15 mm in length are preserved (Figs. 3 and 4). In addition, examples of triclinic crystal termination are also seen in phase 1 (Fig. 3). Phase 2 consists of dark‐toned patches, which appear as interstitial material between phase 1 grains. The size of the interstitial areas varies significantly, with some areas of phase 2 material extending to over 1 cm in diameter (Fig. 3) while others are less than 1 mm. In MAHLI images (Fig. 3), phase 2 appears to have a fine‐grained texture with multiple components of slightly varying dark tone.

Two other phases are significantly less abundant: phase 3 is a gray/brown‐toned phase that occurs between phase 1 crystals. Phase 4 is a light‐toned phase (brighter than phase 1) and observed as small veins around phase 2, occupying small interstitial areas. One of the notable features of the Askival texture is the presence of mm‐sized and smaller pits (Figs. 3 and 4). These are sometimes seen to be lined with a layer of light brown dusty material. The origin of these and the relationship to alteration are discussed in a later section.

Pixel counting of the four phases on MAHLI images (Fig. 4) using Adobe Photoshop indicates that the approximate abundances by visible area of the four phases are as follows: 64% phase 1, 30% phase 2, 5% phase 3, and 1% phase 4.

#### Bindi

Bindi is an angular, blocky rock, approximately 5 cm in diameter. The RMI image of Bindi (Fig. 5a) shows two distinct phases. Bindi phase 1 consists of subhedral and euhedral light‐toned ≤1 cm crystals, comprising approximately 80% of the rock (Cousin et al., 2017). Crystals appear to be better formed than those seen in Askival phase 1, with clear elongated tabular crystals, in some cases aligned to give a roughly planar fabric. The remaining 20% of the resolvable texture consists of phase 2, a dark interstitial phase. Further subdivision of phase 2 is not possible with the imagery available. The high proportion of one phase and roughly planar fabric are consistent with an origin as a cumulate (Cousin et al., 2017).

### Elemental Composition

#### Askival

The Askival target was sampled using three separate LIBS rasters: Askival (10 × 1), Askival_2 (3 × 1), and Askival_3 (10 × 1), the positions of which are shown in Fig. 5b–d. The Askival and Askival_3 rasters were performed with the typical 30 laser analyses per point, whereas Askival_2 used an extended sequence of 150 laser analyses. Of the total of 23 sampling points between the three rasters, 15 have sampled the Phase 1 light‐toned phenocrysts, three have sampled the phase 2 dark‐toned assemblages, one has analyzed the phase 4 white veins and border of dark material, only one point has sampled the green/brownish phase 3 (as an individual grain, in the border), and three have sampled the interface between phases 1 and 2. Unfortunately, there is no sampling point on the greenish/brownish material located at the border of the dark assemblages.

**Fig. 6:**
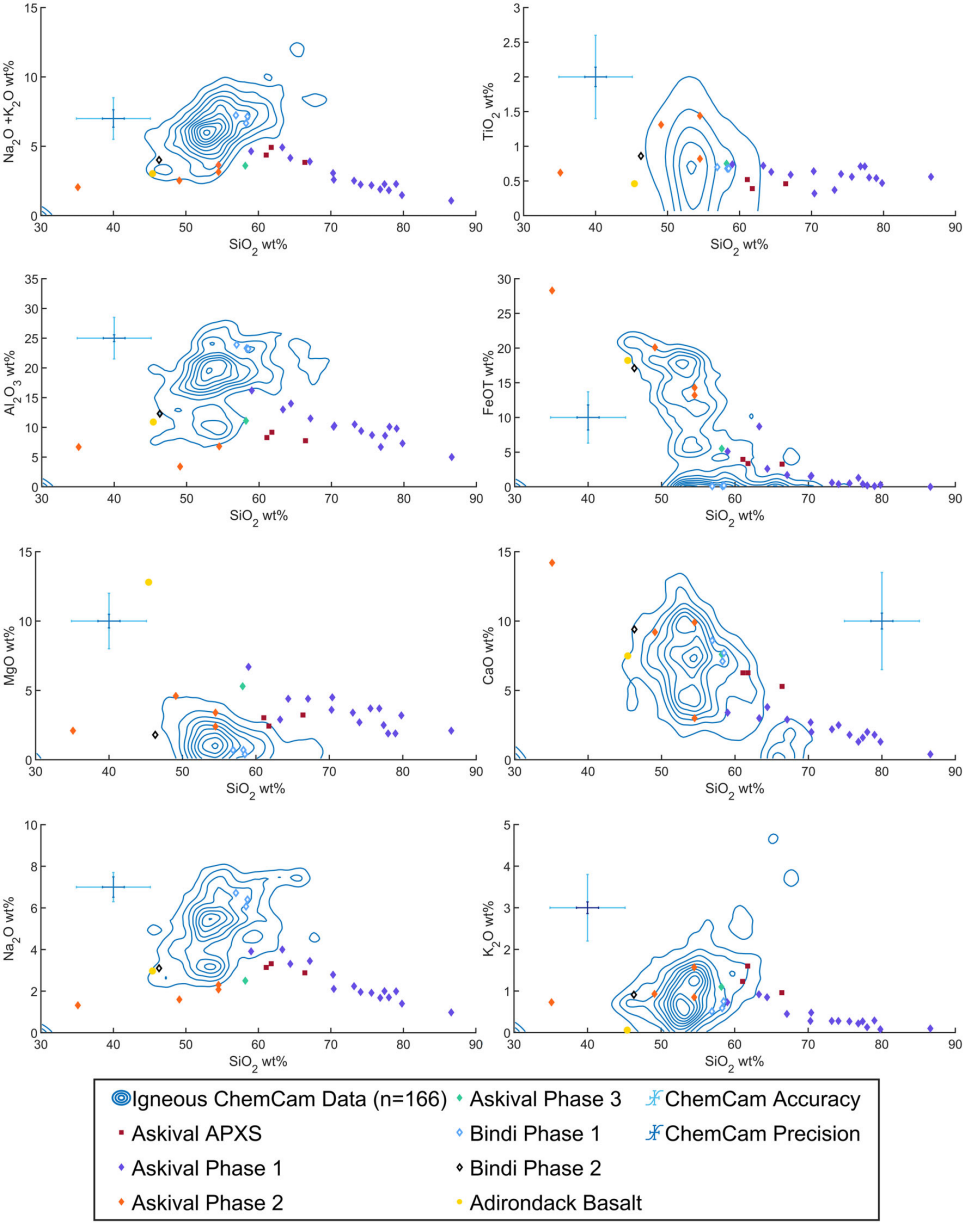
Major oxide plots for Askival, Bindi ChemCam, and Askival APXS data. Igneous contours represent bulk ChemCam data (Edwards et al., 2017). Askival's light‐toned phase 1 shows an enrichment of silica correlating with a decrease in other elements, representing an alteration trend away from Bindi's composition. This is shown in more detail in Fig. 8. Adirondack basalt composition from McSween et al. (2004). Error bars show ChemCam precision and accuracy from Bedford et al. (2019). (Color figure can be viewed at wileyonlinelibrary.com.)

Major oxide compositions are provided in Table 1, from which a clear distinction can be seen between Askival's four texturally distinct phases. We also plot these phase compositions, with the exception of phase 4 (discussed below), on a set of Harker plots (Fig. 6) alongside bulk ChemCam data from igneous and sedimentary samples from Gale crater, as well as a meteorite composition and the Adirondack class basaltic composition from Gusev crater for comparison.

**Table 1:** Askival ChemCam oxide measurements.

Raster	Point	Phase	SiO_2_ (wt%)	TiO_2_ (wt%)	Al_2_O_3_ (wt%)	FeO_T_ (wt%)	MgO (wt%)	CaO (wt%)	Na_2_O (wt%)	K_2_O (wt%)	Total (wt%)
Askival	1	2	35.1	0.62	6.7	28.3	2.1	14.2	1.3	0.73	89.2
Askival	2	4	0.1	0.2	0.6	6.3	2.3	30.8	0.7	n.d.	41.1
Askival	3	1	63.3	0.72	13	8.7	2.9	3	4.0	0.92	96.5
Askival	4	1	86.6	0.56	5	n.d.	2.1	0.4	1.0	0.1	95.8
Askival	5	1	67.1	0.59	11.5	1.7	4.4	2.9	3.5	0.45	92.0
Askival	6	2	54.5	1.44	6.8	13.2	2.4	3	2.1	1.57	85.0
Askival	7	2	49.1	1.31	3.4	20.1	4.6	9.2	1.6	0.93	90.3
Askival	8	Interface	42.6	0.81	8.8	10.5	2.5	19	1.7	0.78	86.7
Askival	9	1	74.1	0.6	9.4	0.4	2.7	2.5	2.0	0.28	91.9
Askival	10	1	77.4	0.71	8.6	0.4	2.5	1.6	2	0.27	93.4
Askival_2	1	1	78	0.55	10.1	0.2	1.9	2	1.7	0.13	94.6
Askival_2	2	1	70.3	0.64	10.1	1.5	3.6	2.7	2.8	0.28	91.8
Askival_2	3	Interface	32.6	0.89	11.5	8.3	2	23.3	2.4	0.44	81.3
Askival_3	1	Interface	54.5	0.82	6.8	14.3	3.4	9.9	2.3	0.85	92.9
Askival_3	2	1	59	0.74	16.2	5.1	6.7	3.4	3.9	0.73	95.8
Askival_3	3	1	73.2	0.37	10.5	0.6	3.4	2.2	2.2	0.28	92.8
Askival_3	4	1	70.4	0.32	10.3	1.6	4.5	2	2.1	0.48	91.7
Askival_3	5	1	79.8	0.47	7.3	0.3	3.2	1.3	1.4	0.08	93.8
Askival_3	6	1	75.6	0.56	8.7	0.5	3.7	1.8	1.9	0.27	93.0
Askival_3	7	1	76.8	0.71	6.7	1.3	3.7	1.3	1.7	0.22	92.4
Askival_3	8	1	79	0.54	9.8	0.1	1.9	1.8	2.0	0.29	95.5
Askival_3	9	3	58.2	0.75	11.1	5.5	5.3	7.6	2.5	1.1	92.0
Askival_3	10	1	64.4	0.63	14	2.6	4.4	3.8	3.3	0.85	94.0
Reference compositions											
Plagioclase An_40_	–	–	58.0	–	26.5	–	–	8.3	6.33	0.87	100
Ferrohastingsite	–	–	36.4	–	10.3	37.0	–	11.3	3.13	–	98.18

**Fig. 7:**
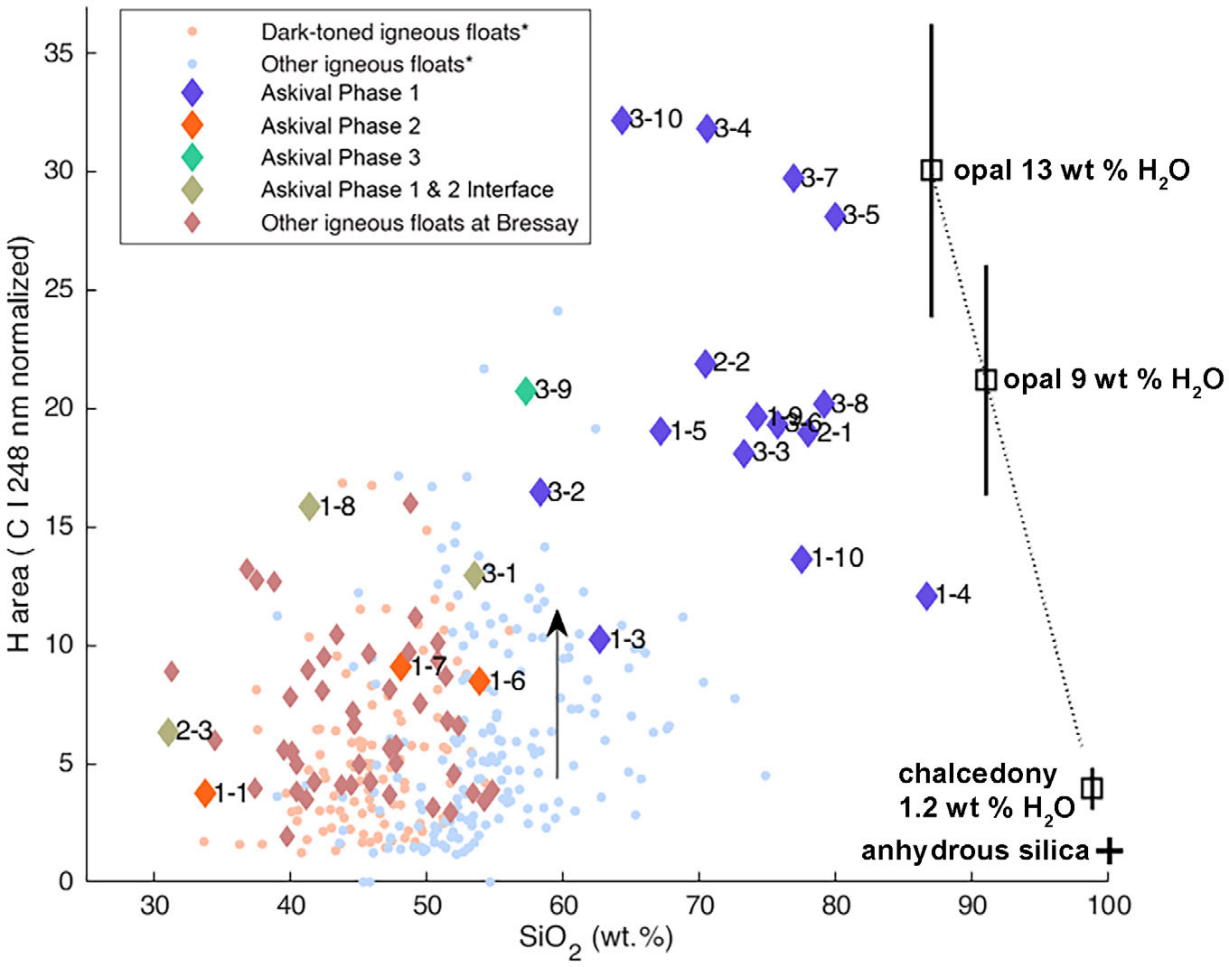
Scatter plot showing normalized hydrogen peak area against SiO_2_ content. Data are plotted for all ChemCam points in Askival as well as other igneous material from the Bressay locality and across the Bradbury sedimentary group. Data point annotations indicate raster (first number) and point number (second number). The vertical arrow illustrates the bias from random enhancement in hydrogen signal related to the roughness of the target surface (Rapin, Bousquet, et al., 2017). The black cross to the lower right of the plot represents ChemCam limit of detection for water for anhydrous silica (about 0.2 wt%), and black squares show endmembers for chalcedony, microcrystalline quartz which may contain 1 wt% water (Flörke et al., 1982), and example of opals with various possible water contents. The dotted line represents calibration to opaline silica, with 1‐sigma uncertainty related to the laboratory calibration and instrument response function correction factor (Rapin et al., 2018; Rapin, Meslin, et al., 2017). *Igneous float rocks from Bradbury described in Cousin et al. (2017). (Color figure can be viewed at wileyonlinelibrary.com.)

**Fig. 8:**
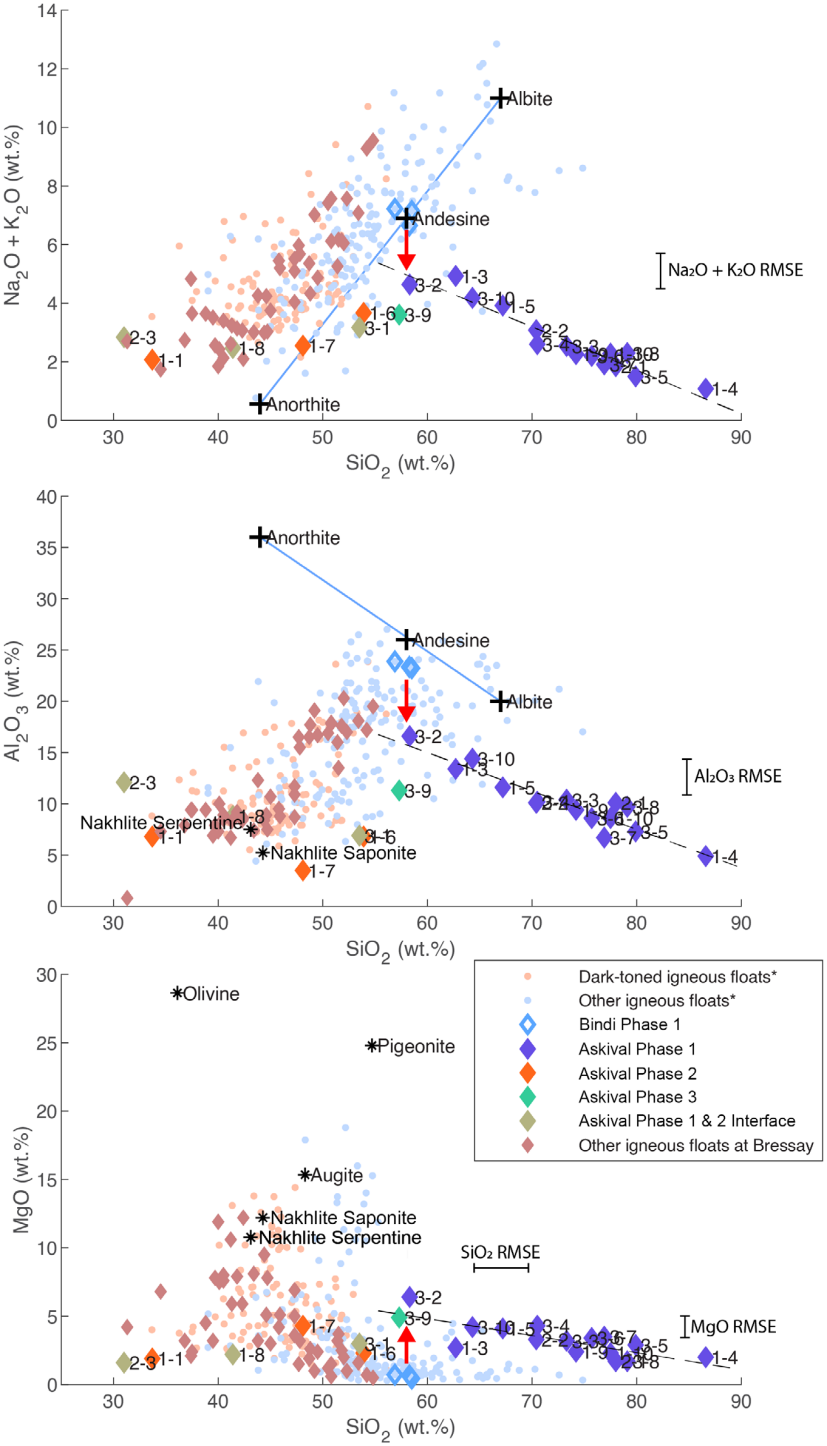
Scatter plots of total alkali, Al_2_O_3_, and MgO versus silica showing linear projections of stoichiometric plagioclase feldspar (Or_05_, blue line) as well as chemical trends due to modeled linear dissolution of feldspar (loss of alkali and aluminum) and enrichment in Mg (red arrow) from a Bindi‐like plagioclase starting composition. It then shows the silicification trend highlighting the enrichment in silica of the altered feldspar (dashed line). Nakhlite serpentine composition as reported (Hicks et al., 2014; Piercy et al., 2022). *Igneous float rocks from Bradbury described in Cousin et al. (2017). (Color figure can be viewed at wileyonlinelibrary.com.)

Phase 1 (Table 1) is highly enriched in SiO_2_ (58–86.6 wt%) but relatively low in Al_2_O_3_ (4.9–16.6 wt%), CaO (0.5–3.9 wt%), and alkalis (<5 wt%). FeO is low (<8.7 wt%) as well as MgO (<7 wt%)—but both are variable. The Cl spectral line is observed in all of these points (see Appendix S1). Phase 1 also exhibits a stronger H signal compared to all the other phases observed in Askival (Fig. 7). The silica and hydrogen enrichments are close to an opal endmember with 9 wt% H_2_O, and some points (Askival_3 #4, #5, #7, and #10) having an even stronger signal, corresponding to around 13 wt% of H_2_O, with slightly lower SiO_2_ but increases in other elements (Fig. 8) which could be related to the combined presence of other hydrated minerals in this phase.

Phase 2 (Table 1) has a variable chemistry. Three points have sampled the interface between phase 2 and phase 1 (Askival #8, Askival_2 #3, Askival_3 #1). The CaO content in these three points is >10 wt%, and also varies with depth in most cases. These three points enriched in CaO exhibit the S lines and Cl lines (see Figs. S1–S3), indicating that as well as a mix of phases 1 and 2 these points may also be sampling CaSO_4_ at the phase boundary. Askival #1 also contains a high proportion of CaO despite not being visibly placed at the boundary of two phases.

Excluding the mixed phase points from our analysis, phase 2 contains a moderate amount of SiO_2_ (35.1–54.5 wt%); it is relatively poor in Al_2_O_3_ (<3.4–6.8 wt%) and MgO (2.1–4.6 wt%) but contains a higher FeO content (13.2–28.4 wt%) as well as K_2_O (0.7–1.6 wt%) compared to phase 1. The CaO content varies from 3 to 14.2 wt%, with the highest CaO content corresponding to Askival #1.

**Fig. 9:**
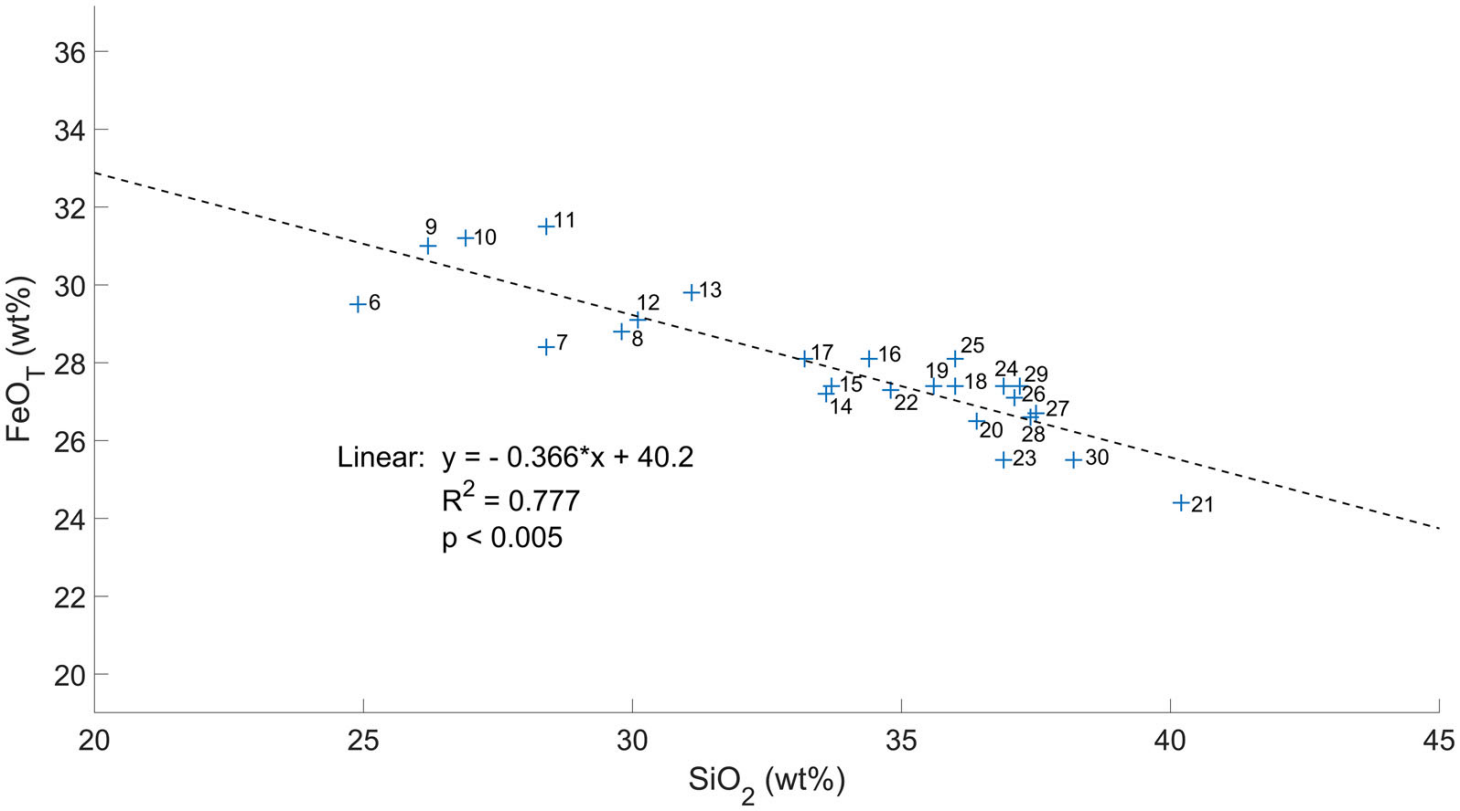
Shot‐to‐shot analysis of FeO_T_ and SiO_2_ content at target point Askival #1. Number labels indicate shot order, first five shots are excluded due to dust cover. Linear regression indicates a negative correlation between FeO_T_ and SiO_2_, with earlier points (6–13) having higher FeO_T_ and lower SiO_2_. (Color figure can be viewed at wileyonlinelibrary.com.)

Askival #1 also shows the highest FeO content with the lowest SiO_2_ content (<35 wt%—Table 1). Shot‐to‐shot analysis of Askival #1 (Fig. 9) shows an anticorrelation between SiO_2_ and FeO. Askival #6 and #7 are enriched in Ti at 1.3 and 1.4 wt%, respectively, and contain the highest K_2_O content for this class (0.9 and 1.6 wt%, respectively). These two points also exhibit the CaF molecular line, which means that they contain at least 0.2 wt% F (Forni et al., 2015). Other points from the dark‐toned material could contain some F but below or close to the limit of detection.

All six points that wholly or partially sampled phase 2 are enriched in Cr and Mn compared to other points (see Appendix S1). Cr is particularly elevated in Askival #6 and #7, and Mn in Askival #1, #6, #7, and Askival_3 #1. These points are also enriched in Rb compared to phase 1, probably due to their relatively high K_2_O content. This dark‐toned material shows the lowest H signal among phases sampled on Askival. Nevertheless, this dark‐toned material has the same hydration level as previous igneous rocks observed at Gale (Fig. 7).

Phase 3 (Table 1) was only sampled by one point (Askival_3 #9, Fig. 5). Phase 3 has significantly higher Al_2_O_3_ compared to phase 2, at 11.3 wt% compared to 6.8 and 3.5 wt% in points Askival #6 and #7, respectively. It contains a moderate amount of SiO_2_ and CaO. FeO is depleted and lower than that of phase 2 (5.4 wt%) whereas this point shows one of the highest MgO contents of the data set (4.9 wt%). As observed in Askival #6 and #7 (phase 2), the phase 3 K_2_O content is relatively high (1.1 wt%). S and Cl were detected, whereas the F signal seems to be observed but is close to the limit of detection (see Appendix S1). The H signal is elevated, similar to phase 1, with an equivalent 9 wt% of H_2_O (Fig. 7).

Phase 4 (Table 1) displays extremely high CaO and low SiO_2_, with very low abundances for all other quantified oxides, leading to a lower oxide sum than the other sampled phases. The only point that has sampled the light‐toned phase 4 material (Askival #2) shows the highest CaO content in the data set (31 wt%). Analyzing the 30 individual laser shots, we can observe that the CaO content increases with depth, up to 35.9 wt% on the last shot. This point also shows the highest S detection observed on Askival (see Appendix S1).

**Fig. 10:**
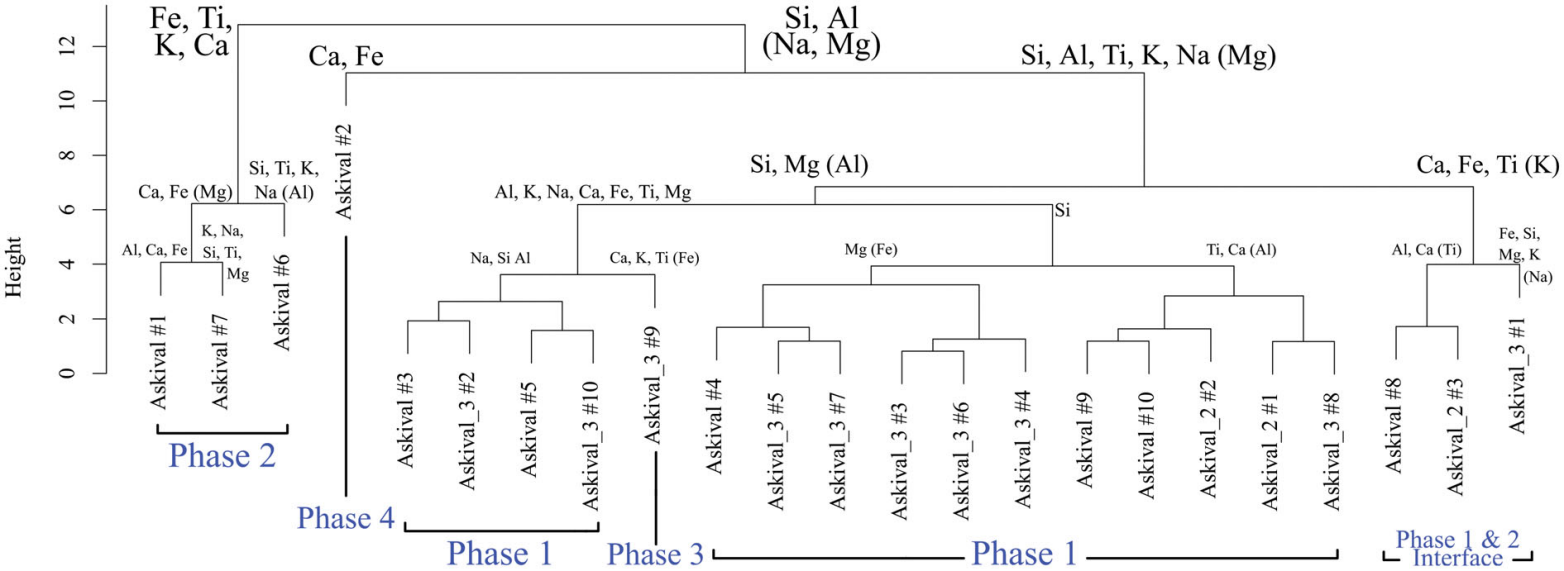
Independent component analysis dendrogram displaying hierarchical clustering of Askival target points from all three rasters, based on major oxide composition. Elemental labels on each branch show the components which are higher in that cluster, with parentheses indicating that there is significant overlap in an element between the two clusters at that dividing step. Blue text phase classification based on RMI images (see Fig. 3). A clear compositional distinction between texturally identified phases 1–3 is present. The light‐toned phase is divided into two halves, primarily differing in SiO_2_ content (see the section Askival). (Color figure can be viewed at wileyonlinelibrary.com.)

The ICA dendrogram (Fig. 10) produced from the three Askival ChemCam rasters separates the phases into groupings which correspond well to the visually and compositionally identified phases described above, with the exception that the light‐toned phase 1 points are partitioned into two clusters which vary primarily in the extent of their SiO_2_ enrichment. One group contains all phase 1 points which have SiO_2_ contents <70 wt% and the other contains all points with SiO_2_ content >70 wt%. The former group is also associated in the dendrogram with the single point Askival_3 #9, although a clear compositional distinction is present between the two from CaO content, which is at 7.6 wt% in phase 3 whereas the highest CaO in the associated phase 1 points is 3.8 wt%. There is also raised K_2_O (1.1 wt%) and lower Na_2_O (2.4 wt%) in the Askival_3 #9 point compared to this set of phase 1 points. We will therefore continue to classify the light‐toned material in Askival as a single phase (phase 1), but this analysis highlights the extent of variation in silicification of this phase.

#### APXS Analyses of Askival

The APXS instrument field of view (~1.7 cm) means that the analyses of Askival represent a mixed composition of the phases identified, with contributions from phases 1, 2, and 4. The placement of the APXS analyses avoids any visually identified phase 3 material. Taking these analyses as representing an average composition of Askival, we compare them to a modally calculated ChemCam composition (Table 2; Fig. 6), which was calculated using the abundances of each phase as measured on MAHLI images (64% phase 1, 30% phase 2, 5% phase 3, and 1% phase 4), and the average composition of each phase across all ChemCam LIBS analyses. Similar calculations were also performed for Bindi, using 85% phase 1 and 15% phase 2 material.

**Table 2:** Askival APXS oxide measurements.

	SiO_2_ (wt%)	TiO_2_ (wt%)	Al_2_O_3_ (wt%)	FeO_T_ (wt%)	MgO (wt%)	CaO (wt%)	Na_2_O (wt%)	K_2_O (wt%)	SO_3_ (wt%)	Total (wt%)
Askival_raster1^a^	66.4 ± 1.0	0.46 ± 0.05	7.8 ± 0.3	3.3 ± 0.1	3.2 ± 0.2	5.3 ± 0.1	2.9 ± 0.2	1.0 ± 0.1	7.7 ± 0.3	97.9
Askival_raster2^a^	61.8 ± 0.6	0.39 ± 0.02	9.2 ± 0.2	3.4 ± 0.1	2.4 ± 0.1	6.3 ± 0.1	3.3 ± 0.1	1.6 ± 0.1	9.7 ± 0.1	98.0
Askival_raster3^a^	61.1 ± 1.0	0.52 ± 0.05	8.3 ± 0.3	4.0 ± 0.1	3.0 ± 0.2	6.3 ± 0.1	3.1 ± 0.2	1.2 ± 0.1	9.8 ± 0.3	97.3
Calculated modal composition	68.6	0.80	9.4	8.3	3.7	5.6	2.4	0.7	0.61	100

a Gellert, R., Mars Science Laboratory Alpha Particle X‐Ray Spectrometer RDR Data V1.0, MSL‐M‐APXS‐4/5‐RDR‐V1.0, NASA Planetary Data System, 2013.

Comparing this to the APXS analyses, which measure 61–68 wt% SiO_2_ in Askival, implies approximately a 6–10% increase in silica from a modally calculated composition for Bindi, despite Bindi having greater (85%) felsic material in our modal calculations, which would increase the silica abundance in a pristine feldspar cumulate sample.

There are, however, some points of disagreement between the APXS analyses and the ChemCam LIBS results. Primarily, APXS analysis includes SO_3_, which is excluded from ChemCam analysis. We include some SO_3_ in our modal calculations from ChemCam results in the form of phase 4, the calcium sulfate phase, which is added using an assumed pure CaSO_4_ molecular formula rather than directly from sample analysis. However, this does not account for the quantity of SO_3_ measured by APXS, which is approximately 8% higher across the analyses than in our calculations.

Terrestrial experiments with an analog instrument have shown that the APXS system is prone to overestimating the sulfur component present in sulfate–silicate mixtures by approximately 1.5 times (Berger, Schmidt, et al., 2020), which is of particular relevance in the case of an SiO_2_‐enriched sample such as Askival. Furthermore, the SO_3_ measured by APXS scales proportionally with the CaO content in the three analyses, consistent with a calcium sulfate source. As such, it seems likely that the APXS CaSO_4_ measurement is somewhat overestimated in line with Berger, Schmidt, et al. (2020), but still indicates that there may be additional calcium sulfate deposited on the surface of Askival that is not clearly visible in the MAHLI or RMI images. Although the SO_3_ is not systematically quantified with ChemCam, this is also consistent with the total weight percentages throughout Askival's LIBS analysis being below 100%.

Other major elements have a variety of agreement between APXS and our modal calculations from ChemCam results. Al_2_O_3_, MgO, and alkali elements are generally close to matching although K_2_O varies significantly between APXS analyses and only the lower end of this range is close to the modal calculations.

However, our modal calculation predicts significantly higher FeO content in Askival's composition than the APXS analyses show. Comparing the high FeO points in phase 2 to igneous geochemistry from other targets in Gale crater (Fig. 5), we see that Askival #1's FeO content is significantly above the usual range and shot‐to‐shot data from this point (Fig. 9) indicate an iron oxide component is present. As there are only four points sampling phase 2 to any extent from ChemCam data, our modal analysis may be overestimating the FeO content due to this outlier composition, as compared to the wide field of view analysis performed by APXS.

#### Bindi

Bindi was sampled using a single 5 × 1 LIBS raster, leading to a less extensive sampling of its chemistry than of Askival. However, all phases identified from RMI imagery are sampled by at least one LIBS point. In contrast to Askival, the equivalent light‐toned phase 1 in Bindi shows very little variation in SiO_2_ (Fig. 6), or any other major elemental oxides (Table 3), indicating that it did not undergo the same postformation silicification process. This is reinforced by the near‐stoichiometric An_38_‐An_40_ plagioclase feldspar composition seen in this Bindi phase 1 (Table 3). Target points 3 and 5 have higher mafic oxide abundances but retain relatively high Al_2_O_3_ and in the case of point 3, similar to SiO_2_. In combination with the ChemCam targeting RMI (Fig. 5a) which shows point 3 located at a boundary between phase 1 and the more mafic phase 2, we classify point 3 as representing a linear combination of the two phases and point 5 as being representative of phase 2 chemistry in Bindi.

**Table 3:** Bindi ChemCam oxide measurements.

Target	Point	Phase	SiO_2_ (wt%)	TiO_2_ (wt%)	Al_2_O_3_ (wt%)	FeO_T_ (wt%)	MgO (wt%)	CaO (wt%)	Na_2_O (wt%)	K_2_O (wt%)	Total (wt%)
Bindi	1	1	58.3	0.68	23.3	0.1	0.7	7.1	6.1	0.59	96.8
Bindi	2	1	56.9	0.7	23.9	0.1	0.7	8.6	6.7	0.51	98.1
Bindi	3	1 and 2	56	0.89	18.1	9.9	1.4	7.5	4.2	1.6	99.6
Bindi	4	1	58.5	0.68	23.2	0.2	0.4	7.7	6.4	0.75	97.9
Bindi	5	2	46.3	0.86	12.3	17.1	1.8	9.4	3.1	0.91	91.8

## Interpretation and Discussion

### Mineralogical Assessment

The LIBS technique gives chemical information about the different phases of the target; their mineralogy can be assessed by combining both the chemistry and visual observations. It is usually easier for feldspars as their shape, texture, and chemistry are easily distinguished. It is more challenging to distinguish the mafic phases, as they have less distinct shapes and colors in the RMI and MAHLI images. Also, their chemistry can vary significantly among each mineral. In the specific case of Askival, the mineralogical assessment is even more complex as the chemical and visual analyses suggest that this sample has recorded at least one and possibly several hydrous alteration overprints.

Combining the chemical data with the textural description in the section Elemental Composition, we identify phase 4 as calcium sulfate‐bearing points (measurement of sulfur with ChemCam presents a number of difficulties, discussed in Dyar et al. [2011], and therefore is not included in our elemental oxide measurements here). Phase 1 has a texture and color consistent with plagioclase feldspar. However, the variably high SiO_2_ content (Fig. 6; Table 1) means that phase 1 does not present a stoichiometric feldspar composition. In addition, the negative correlation between major elements including Al_2_O_3_ and SiO_2_ shown in Fig. 6 trends away from stoichiometric albite–anorthite compositions (Fig. 8). We interpret this as evidence of postcrystallization alteration, which makes Askival's composition harder to interpret from a purely magmatic perspective. We consider alkali remobilization and silica enrichment in more detail in a later section.

Half of the phase 2 points are enriched in Ca and have an S detection. These points were located on the border of the dark‐toned material, which means they could correspond to a mixture between phase 1, phase 2, and phase 4. Here, we will focus our mineralogical assessment on points that are not mixed with a CaSO_4_ component.

Phase 2 has a more variable chemistry than phase 1, but broadly consists of more FeO_T_‐ and MgO‐rich minerals. SiO_2_ varies by almost 20 wt% across the three points classified as phase 2, correlating positively with increased H_2_O peak area (Fig. 7). It is unclear if this hydration is native to the mineral compositions or is the result of the same postformation alteration event that increased the silica content of phase 1. Point #1 in the Askival raster has the lowest SiO_2_ content of phase 2. Examination of the individual LIBS shots on this point reveals higher FeO and CaO contents in early shots, with FeO being anti‐correlated with SiO_2_ indicating the presence of iron oxide and calcium sulfate grains in the shot area.

Three phase 2 points do not seem to be mixed with the highly altered phase 1 or sulfates: Askival #1, Askival #6, and Askival #7. However, their chemistry does not match with any pure mafic mineral such as olivine (SiO_2_, CaO, Al_2_O_3_, and alkalis are all too high) or pyroxenes (Al, Ca, and alkalis are too high, Mg is too low relative to Fe). MAHLI images (Figs. 3 and 4) also reveal some variation in the tone across regions identified as phase 2, supporting interpretation as a fine‐grained mixture of mafic minerals.

The presence of amphiboles or clays is a possibility suggested by detection of F in these points, as well as their relatively high Al and K contents. The hydration state of these two points is relatively low (compared to the light‐toned material), but still around 2 wt% H_2_O, which is consistent with such phases. Clays and amphiboles cover such a wide variety of compositions that it is not possible to test all the possibilities. Several normative calculations were made, taking into account different types of phases: oxides (ilmenite), feldspars using albite and/or orthoclase, pyroxenes with wollastonite, ferrosilite and enstatite, saponite (composition as observed in nakhlite meteorite alteration veins, see Hicks et al., 2014), amphibole (Ca‐Mg and Ca‐Mg‐Fe hornblende, actinolite), and quartz. Results are listed in Table 4. The best match was obtained using a mixture dominated by pyroxenes (ferrosilite, wollastonite, enstatite—45 mole% for Askival #6 and 85 mole% for Askival #7), albite (5 and 4 mole% resp.), ilmenite (3 mole%), saponite (4 and 0 mole% resp.), and some quartz (44 mole% for Askival #6 and 8 mole% for Askival #7). The presence of saponite is consistent with the detection of F in #6. Phase 3, which was only sampled in one LIBS point, has a unique chemistry compared to the other phases, with intermediate SiO_2_ and FeO_T_, high Al_2_O_3_, CaO, and K_2_O. A similar approach as for phase 2 was performed by doing some normative calculations (Table 4).

**Table 4:** Mafic phase normative compositions.

Target	Point	Ilm^a^ (mole%)	Ab^a^ (mole%)	Sap^a^ (mole%)	Hbl^a^ (mole%)	Fs^a^ (mole%)	Wo^a^ (mole%)	Qz^a^ (mole%)	Mag^a^ (mole%)	En^a^ (mole%)	Or^a^ (mole%)	An^a^ (mole%)	Msc^a^ (mole%)
Askival	#1	1.2	3.3	2.7	1.1	n.d	33.3	n.d.	58.4	n.d.	n.d.	n.d.	n.d.
Askival	#6	2.9	5.5	3.5	n.d.	26.7	8.6	43.6	n.d.	9.2	n.d.	n.d.	n.d.
Askival	#7	2.6	4.1	n.d.	n.d.	40.3	26.3	8.4	n.d.	16.8	1.6	n.d.	n.d.
Askival_3	#9	1.9	7.9	3.8	2.4	n.d.	17.5	66.6	n.d.	n.d.	n.d.	n.d.	n.d.
Bindi (a)	#5	2.4	11.1	n.d.	1.8	36.9	29.7	n.d.	13.4	2.5	n.d.	n.d.	2.1
Bindi (b)	#5	2.1	9.8	n.d.	n.d.	27.3	20.9	n.d.	17.3	8.8	1.9	12.0	n.d.

Bindi (a) calculation was performed using hornblende and muscovite endmembers in place of orthoclase and anorthite, which Bindi (b) includes.

a International Mineralogical Association abbreviations from Whitney and Evans (2010).

Askival_3 #9 is a distinct data point within phase 3. This type of material has been observed around most of the dark material, but also as a whole—that is the case for Askival_3 #9, but we have sampled the border only by LIBS. From the images (Figs. 3 and 4), this type of texture and morphology suggests that phase 3 could be a result of the alteration of the dark material phase 2. As noted above, these dark phases in Askival #6 and #7 have shown to be a mixture, likely including pyroxenes.

Askival_3 #9 has an Al_2_O_3_ content of 11.1 wt%, higher than either Askival #6 (6.8 wt%) or #7 (3.5 wt%), whereas its FeO_T_ content is significantly lower (5.4 wt% compared to 13.2 wt% and 19.6 wt%, respectively). As such, our normative calculations of this observation point could be a mixture of silica (66.6 mole%), wollastonite (17.5 mole%), hornblende (2.4 mole%), albite (7.9 mole%), saponite (3.8 mole%), and ilmenite (1.9 mole%).

### Aqueous Alteration

Numerous compositional features of Askival are beyond the expected chemistry for purely igneous production of a feldspathic cumulate, as exhibited by Bindi. The silicification of the primary feldspar phase, leaching of aluminum and alkalis, and remobilization of magnesium, as well as the increased hydration signal, point to extensive alteration under aqueous conditions. Elevated temperatures trigger the transformation of opal into crystalline, anhydrous quartz (Williams et al., 1985), even with little to no burial (Lynne et al., 2005). Therefore, silicification of Askival most likely did not occur in high‐temperature conditions. Instead, it formed at relatively low temperatures as evidenced by the amount of water found in the altered light‐toned phase at near opal composition (Fig. 7).

The alteration seen in Askival bears some geochemical similarity with alteration halos observed in lacustrine mudstone of the Murray sedimentary unit and overlying eolian sandstone of the Stimson sedimentary unit. At the Marias Pass, Williams, and Bridger Basin locations, silica‐enriched material is associated with fractures that cross the unconformity between the two units (Frydenvang et al., 2017). Yen et al. (2017) and Hausrath et al. (2018) concluded that the presence of these silica‐rich fracture‐associated halos crosscutting bedding in both the Murray and Stimson units is the result of a multiple‐stage alteration process that both passively (through cation leaching) and actively (through precipitation of dissolved silica) enrich the silica content of the constituent minerals. This process would have involved an initial stage of interaction with acidic fluids, with changing pH into neutral to alkaline conditions during precipitation of silica. Neutralization of acidic fluids through contact with basaltic rock types common to Mars has previously been suggested as a result of acidic weathering in Gusev crater (Hurowitz et al., 2006; Zolotov & Mironenko, 2007) and Gale crater (Rampe et al., 2017; Yen et al., 2017).

This occurrence of late, and localized, acidic leaching and remobilization of silica within Gale is a potential route for the Askival float sample to have been silicified. However, unlike the sedimentary outcrops studied in these works, there is no pristine material directly available for comparison to Askival, with Bindi providing our primary example of a possibly similar pre‐alteration rock. Furthermore, geochemical trends in these sedimentary alteration halos indicate that the feldspar grains are more resilient to the silicification process than mafic minerals (Bedford et al., 2020; Hausrath et al., 2018).

**Fig. 11:**
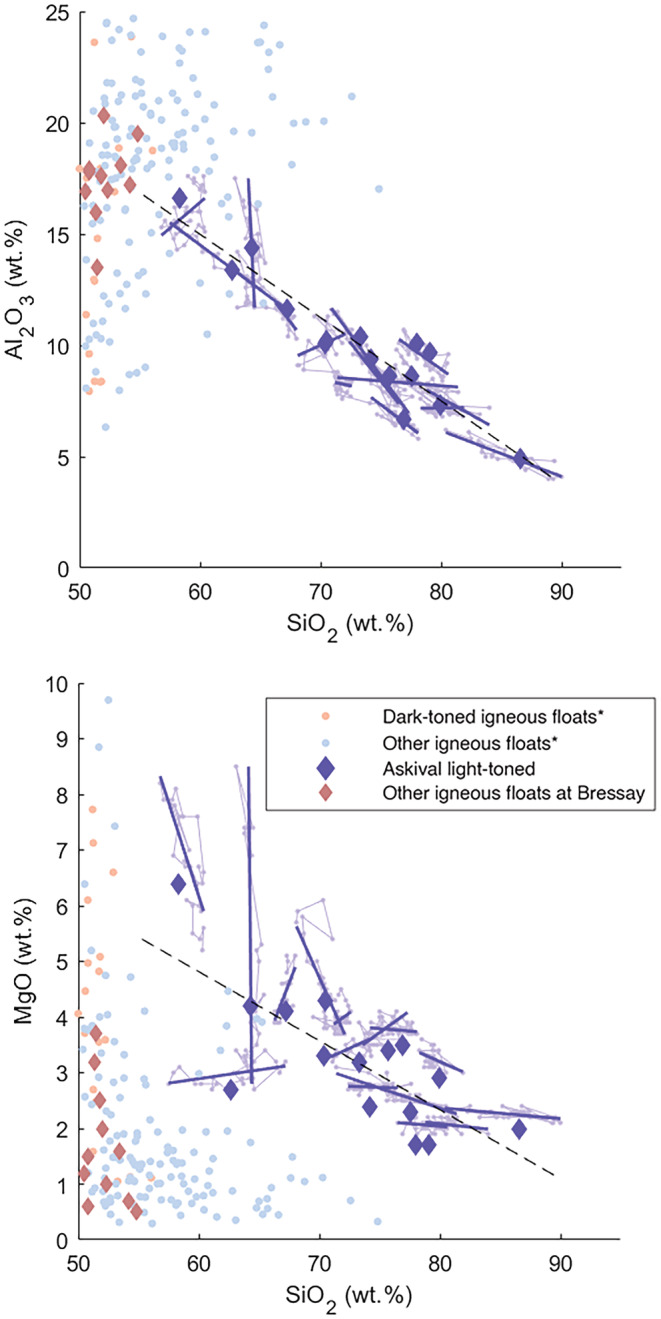
Scatter plots of Al_2_O_3_ and MgO versus silica focusing on Askival phase 1. Shot‐to‐shot composition data (light‐blue dots and lines) are displayed along with shot‐to‐shot linear regression (dark blue lines) for each phase 1 point. *Igneous float rocks from Bradbury described in Cousin et al. (2017). (Color figure can be viewed at wileyonlinelibrary.com.)

The shot‐to‐shot variation on points in the light‐toned phase 1 reveals the complexity of the mixtures observed with several points deviating from a simple opal–feldspar binary mixture, defining a trend which is lower than the trachybasalt Na_2_O, K_2_O, Al_2_O_3_ compositions on the low SiO_2_ end (Edwards et al., 2017) and indicating the possibility of Mg‐enriched phases (Fig. 11). In the absence of a pristine feldspar composition in Askival, the exact remobilization of these elements cannot be quantified, but comparison to Bindi as an equivalent pristine cumulate sample as well as the range of feldspar compositions throughout Gale crater indicates that Askival's feldspar phase would have lost about 25% of its alkali and Al_2_O_3_ content by feldspar dissolution (Fig. 8). Although the proportion of silica enrichment is highly heterogeneous at near mm‐scale as observed by the spread of points along the silica trend, an average 30% increase in silica along with minor Ca‐sulfate re‐creates the phase 1 composition observed by ChemCam. The extensive leaching of aluminum and alkalis from phase 1 is also consistent with acidic alteration (Chardon et al., 2006; Oelkers et al., 1994), which has been hypothesized to be present during the late Noachian and early Hesperian (Berger et al., 2009; Treguier et al., 2008).

Combined Mg and H enrichment specifically on the low SiO_2_ end of phase 1 (Figs. 7 and 8) indicates the presence of an Mg‐bearing secondary mineral phase in a mixture with the silicified feldspars. Clay minerals typically precipitate from solution following feldspar dissolution, forming kaolinite from leached Al and alkali ions (Yuan et al., 2019) and in some cases a further phase of Mg‐rich smectite (Zhu et al., 2006). In the case of Askival, we hypothesize that an Mg‐enriched fluid, either from dissolution of surrounding mafic phases during the acidic alteration or transported from elsewhere, led to the precipitation of an Mg‐phyllosilicate secondary mineral phase on the surface of phase 1. This could have occurred concurrently with the later precipitation of Si as the pH of the fluid evolved into neutral to alkaline conditions.

Phases 2 and 3 are likely relicts of mafic phases that were also partially altered. Whereas the light‐toned crystals are silicified and present a composition consistent with an intimate mixture of feldspars and Mg‐phyllosilicates, the surrounding other phases were in places entirely dissolved as observed with the voids filled by light‐toned Ca‐sulfate (Fig. 4). In the dark‐toned phase 2, the high FeO_T_ point (28.3 wt%) shows shot‐to‐shot Fe to be negatively correlated with silica (Fig. 9), evidence that the iron may be present as Fe‐oxide mixed with relict silicates or silica deposits.

Askival also contains visible small fills of calcium sulfate (phase 4). Such precipitates are common within Gale crater, suggesting that Askival was likely subjected to the same late‐stage alteration identified elsewhere in Gale during the Curiosity traverse and hypothesized to have been the result of late‐stage dissolution of earlier sulfate deposits (Schwenzer et al., 2016).

In summary, we hypothesize that the alteration of Askival began with the hydrolysis of Al, K, Na from the feldspar phase 1, and of Mg from the mafic phase 2, under acidic conditions. Following this, the precipitation of Mg‐bearing clay minerals and additional silica onto the relict feldspar surfaces occurred, producing a surface layer that is heavily enriched in SiO_2_ and has a thin coating of Mg‐phyllosilicate. This stage occurred under low temperatures, preserving the hydration state of the high SiO_2_ layer, and may have been driven by an influx of neutral to alkaline Si‐enriched groundwater, as observed in the diagenetic silica‐enriched halos at Marias Pass (Frydenvang et al., 2017). Finally, Askival was altered by a sulfur‐rich fluid, precipitating the phase 4 calcium sulfate into the voids left behind by prior dissolution, as is seen throughout Gale crater (Schwenzer et al., 2016). This hypothesis is supported by the textural and geochemical measurements made of Askival, but without an in situ example of pre‐alteration minerals, further constraint of the conditions of alteration cannot be made.

The other float samples at the Bressay boulder site do not feature the same increase in hydration signal as Askival across any of their ChemCam analysis points (Fig. 7), indicating that the alteration of Askival took place prior to its emplacement at Bressay.

### Amphibole in Askival?

Our normative calculations and some point analyses such as Askival #1 (once FeO and CaSO_4_ components have been removed) suggest that there may be an amphibole component within the mafic phases. The exact composition of an amphibole is difficult to identify. In our normative calculations (Table 4), we have used a Ca‐Mg hornblende. Phase 3 could also be mainly secondary amphiboles formed from alteration of phase 2 pyroxenes. This seems consistent with the fact that the relict plagioclase grains observed in Askival are also altered by a silicification process. We explore the possible presence and significance of amphibole further with calculated phase relationships relevant to calculated phase stabilities for the Bindi composition at a fixed H_2_O content of 0.5 wt%. As mentioned in the section Normative Calculations, normative identification of mineral phases is not definitive, and our modeling is performed to support the possible presence of amphibole in the mafic phase of Askival.

**Fig. 12:**
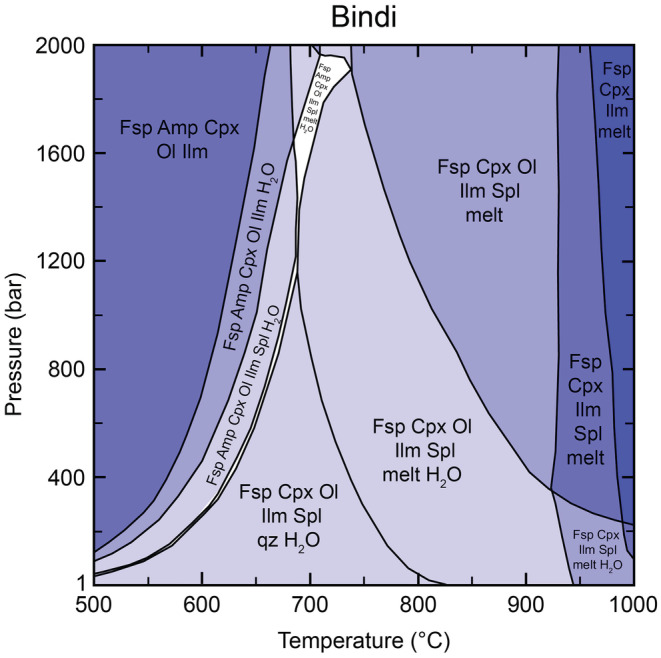
Calculated phase stabilities for the Bindi composition at a fixed H_2_O content of 0.5 wt%. Mineral abbreviations: Amp = amphibole; Cpx = clinopyroxene; Fsp = feldspar; Ilm = ilmenite; Ol = olivine; qz = quartz; Spl = spinel. (Color figure can be viewed at wileyonlinelibrary.com.)

Figure 12 covers the range 1–2000 bars and 500–1000 °C, consistent with an upper crustal setting. The predicted mineral assemblages are dominated by feldspar and clinopyroxene at most conditions, with amphibole stable in the lower temperature range, and melt toward higher temperatures. Feldspar is the most abundant phase with close to 60 vol% in the field where neither amphibole nor melt is stable but decreases in proportion with increasing melt toward higher temperatures (~20 vol% at the highest temperatures). Feldspar at temperatures below the solidus is albite rich (An_50‐60_), while the anorthite component increases with increasing temperature and melt formation due to its higher solidus.

**Fig. 13:**
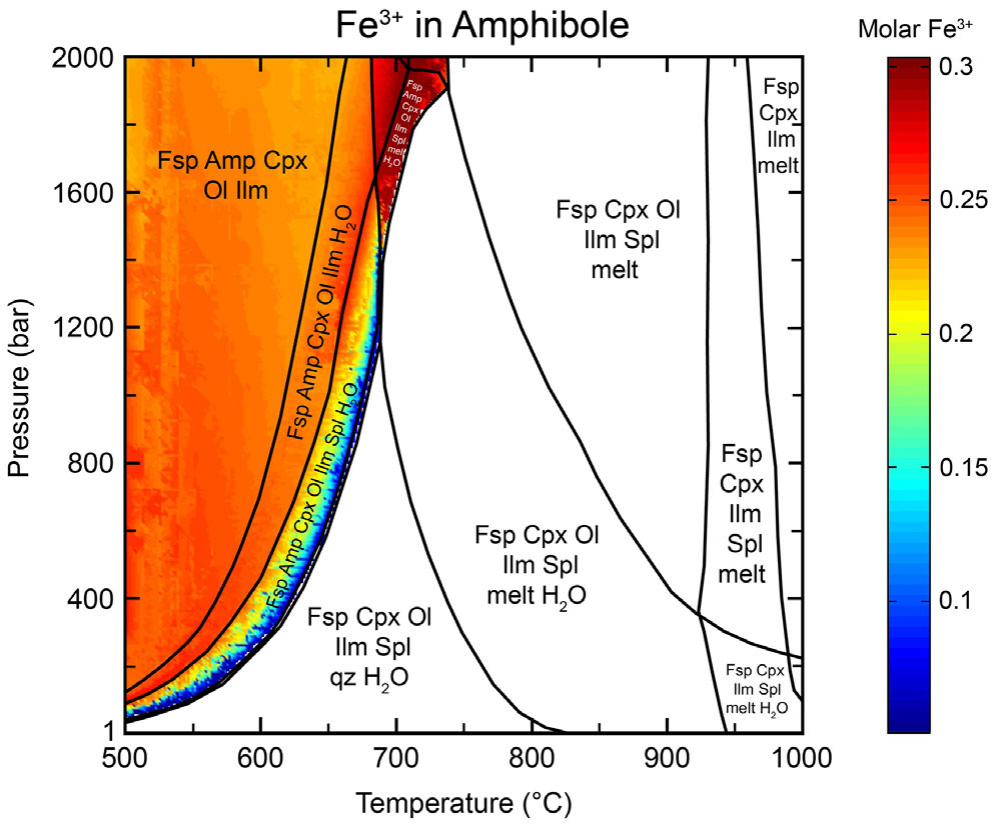
Modeled molar proportions of Fe^3+^ in amphibole for the Bindi composition. Amphibole with the highest Fe^3+^ content coexists with melt at pressures above ~1400 bars. (Color figure can be viewed at wileyonlinelibrary.com.)

Clinopyroxene is present in amounts of 20–30 vol% enriched in diopside component (*X*_Di_. 0.7–0.8). Up to 10 vol% are olivine with *X*_Fa_ >0.8. Amphibole is stable in the lower temperature range up to 740 °C at 2000 bars. At pressures above 1400 bars, amphibole coexisting with melt is the most Fe^3+^ rich (Fig. 13) and is similar in composition to hastingsite (Hawthorne et al., 2012; Locock, 2014). Close to the solidus, modal amounts of amphibole are in the range of 2–7 vol%. Melting starts at temperatures of ~680 °C at 2000 bars and >800 °C at 1 bar. Spinel, ilmenite, and quartz are accessory phases with <2 vol%.

Uncertainties in the modeling derive from uncertainties in the thermodynamic data and solid solution models. The influence of oxides (MnO, Cr_2_O_3_, P_2_O_5_) not considered in the calculation is estimated to be relatively small. Halogens, however, which are currently not included in available solid solution models, have shown to be an important component in Martian meteorites (Filiberto et al., 2014; Giesting & Filiberto, 2016) and amphibole would be the dominant host for Cl and F at conditions considered here (Kendrick, 2019). Experimental and analytical studies have concluded that Cl is preferentially retained in the fluids and therefore more incompatible compared to H_2_O and F (e.g., Fabbrizio et al., 2013; Hecker et al., 2020; Kendrick, 2019), which are therefore expected to have more influence on amphibole stability.

While the water content is fixed to 0.5 wt% in the calculations, higher or lower values do not significantly influence phase stability fields shown in Fig. 12 but may change the phase proportions of hydrous phases such as amphiboles and melt. To focus on amphibole formation, biotite endmembers have been excluded from the calculations. Thermodynamically, biotite is a stable phase at temperatures close to the solidus and amphibole may therefore not be the first OH‐bearing phase to form from melt. However, data and observations of the rock samples do not suggest the presence of biotite, justifying its exclusion from the models.

### Askival–Bindi: Differentiation and Alteration of the Gale Crust

The Gale crust reveals evidence in its igneous float rocks and conglomerate clasts for low‐pressure crystal fractionation, including cumulates, from an Adirondack‐type melt (Edwards et al., 2017). This may also represent early feldspar‐enriched crust on Mars (Sautter et al., 2015). The unaltered Bindi cumulate sample is part of this igneous series. The Askival sample has a more complex history, principally its overprint of silicification and hydration, probably occurring during its residency on the surface of Gale crater or within its near‐surface regolith. Despite that late, intense alteration, particularly of the plagioclase, Askival has preserved a coarse cumulate texture. Although not as clearly defined as the planar cumulate texture in Bindi, the similarity of relict plagioclase alkali ratios (even taking into account some remobilization) to those of the Gale igneous suite (Edwards et al., 2017; Payré et al., 2020) suggests that Askival is an igneous cumulate sample rather than a fragment of a hydrothermal vein.

Amphiboles on Mars are below the detection limits from orbital spectroscopy (Bandfield, 2002) and are absent from mineral assemblages reported from surface missions. However, a lack of orbital detection may not necessarily imply the complete absence of amphiboles. Amphiboles may not be detected due to their relatively low abundance, or because VNIR spectra are typically dominated by one or two phases, thus contributing nonlinearly to the observed spectra (Ehlmann & Edwards, 2014; Ehlmann et al., 2011). This observed lack of amphiboles is in contrast to the Earth, where amphiboles are common in both alteration and magmatic mineral assemblages (Filiberto et al., 2019). However, based on our phase equilibria model, amphibole may be a significant Mars crustal reservoir of volatiles after all, their perceived absence being a product of our as yet limited sampling of the Mars lithosphere by meteorites and landers.

Feldspathic igneous float rocks have been previously observed at Gale and hypothesized to form a significant fraction of the crust on Noachian Mars which was later buried by more basaltic upper volcanic deposits (Sautter et al., 2015). If these feldspathic materials were part of the early crust, it is possible that a significant fraction was not directly buried but exposed to surface or near surface conditions for some time. While Bindi likely represents unaltered buried feldspathic rock, Askival provides a first example of an altered feldspathic rock by fluids that led to the partial dissolution of both felsic and mafic minerals, and the possible formation of Mg‐phyllosilicates (Peretyazhko et al., 2016). Askival represents a possible feldspathic bedrock near‐surface exposure partially altered during that time in Mars history.

## Conclusions

1. Askival and Bindi represent two rare examples of feldspathic cumulate float rocks in Gale crater with >65% relict plagioclase. Bindi appears unaltered and provides a pre‐alteration comparison for Askival, which shows textural and compositional signatures of aqueous alteration and low‐temperature silicification.
2. Askival's alteration occurred through a postformation alteration process, in which localized acidic fluids led to the dissolution and leaching of aluminum and alkali cations from the feldspar phase, followed by precipitation of dissolved silica as the conditions changed to a more neutral pH. This occurred at low temperatures, allowing for the silica‐enriched phase to remain hydrated. Additional silica was likely transported to Askival via neutral to alkaline fluids such as those related to alteration halos at Marias Pass. The acidic fluids also partially dissolved the mafic phase 2 mineral assemblage, remobilizing cations which were incorporated into phyllosilicate secondary minerals and creating voids which were later infilled with Ca sulfate during a more recent late‐stage alteration event, coinciding with similar sulfate precipitation throughout the crater.
3. A presilicification mafic assemblage, interstitial to the relict plagioclase cumulate grains, is present in Askival. Through a combination of LIBS compositional analyses and normative calculations, we suggest that an assemblage of Fe‐Mg silicates including amphibole and pyroxene, Fe oxides, and possibly phyllosilicates may be present although we could not exclude other secondary phases due to the general evidence for alteration in Askival.
4. Phase equilibria modeling of the Bindi composition (which has not been affected by the later silicification) predicts that amphibole and feldspar are stable within an upper crustal setting. This is consistent with the possible presence of amphibole in the parent igneous rocks of Askival and the Martian crust.
5. Compared to Bindi, Askival possibly represents a fragment of an altered feldspathic bedrock at or near the surface in the late Noachian or Hesperian when aqueous alteration was widespread on Mars.

## Supporting information


**Appendix S1.** Normative calculations for Askival and Bini ChemCam analyses.

**Appendix S2.** Askival ChemCam spectra.

## Data Availability

Mars Science Laboratory ChemCam, APXS, MastCam and MAHLI data that support this study are openly available from the NASA Planetary Data System: https://pds‐geosciences.wustl.edu/missions/msl/. Relevant ChemCam derived geochemical data products from this repository are provided in Tables 1 and 3. Relevant APXS derived geochemical data products from this repository are provided in Table 2.
