# Supplementary material for: Askival: An altered feldspathic cumulate sample in Gale crater
Source: Meteorit Planet Sci. 2022 Dec 4;58(1):41–62. doi: 10.1111/maps.13933 (PMC10108227; doi:10.1111/maps.13933)

**Supplementary Information: Askival ChemCam Spectra**

This document contains ChemCam spectral averages from all 3 Askival rasters. Common trace element spectral peak positions are marked with lines, with reference data taken from the U.S. National Institute of Standard and Technology database: <https://www.nist.gov/pml/atomic-spectra-database>

**Supplementary Figure 1: Askival_1 Raster Spectra**


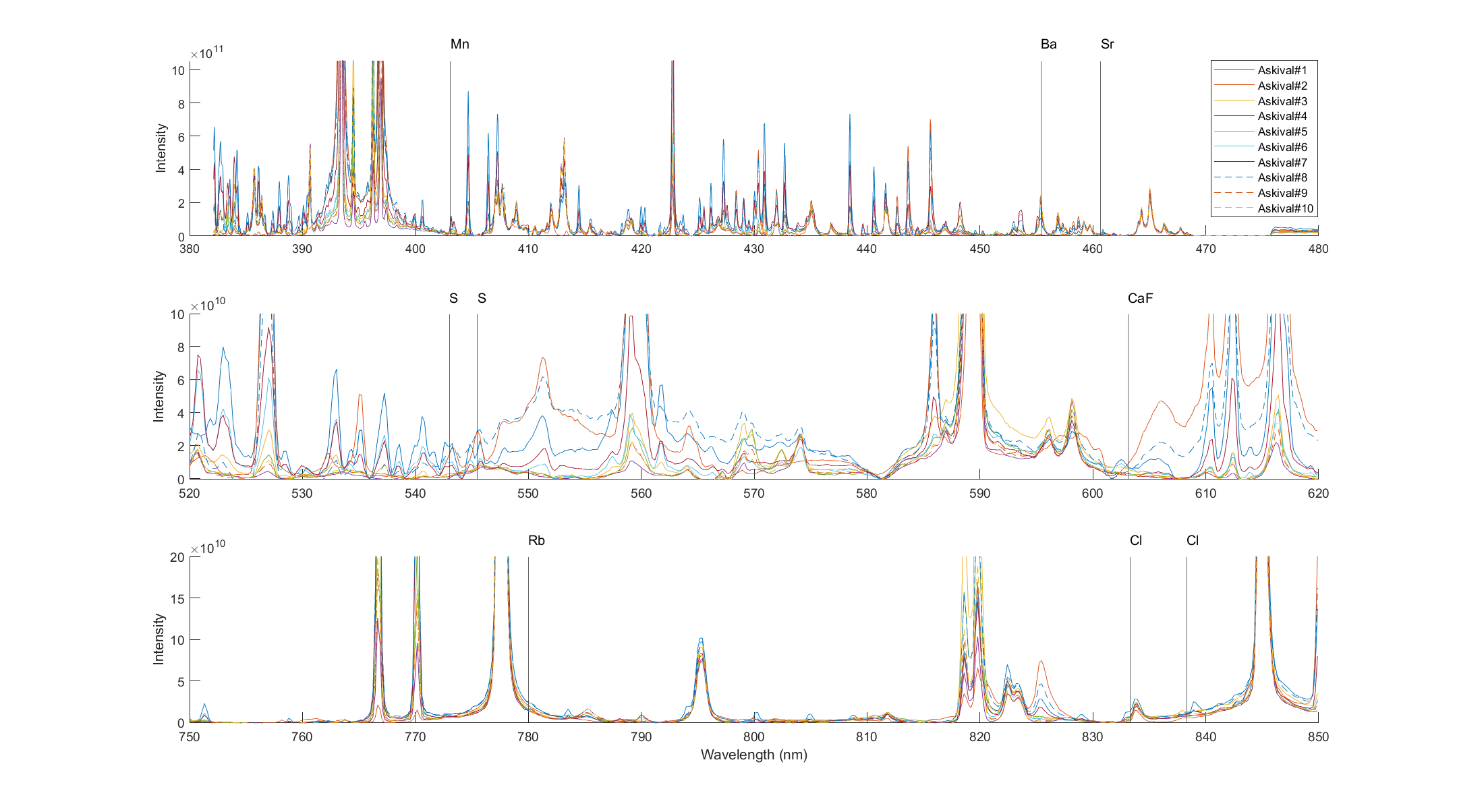


**Supplementary Figure 2: Askival_2 Raster Spectra**


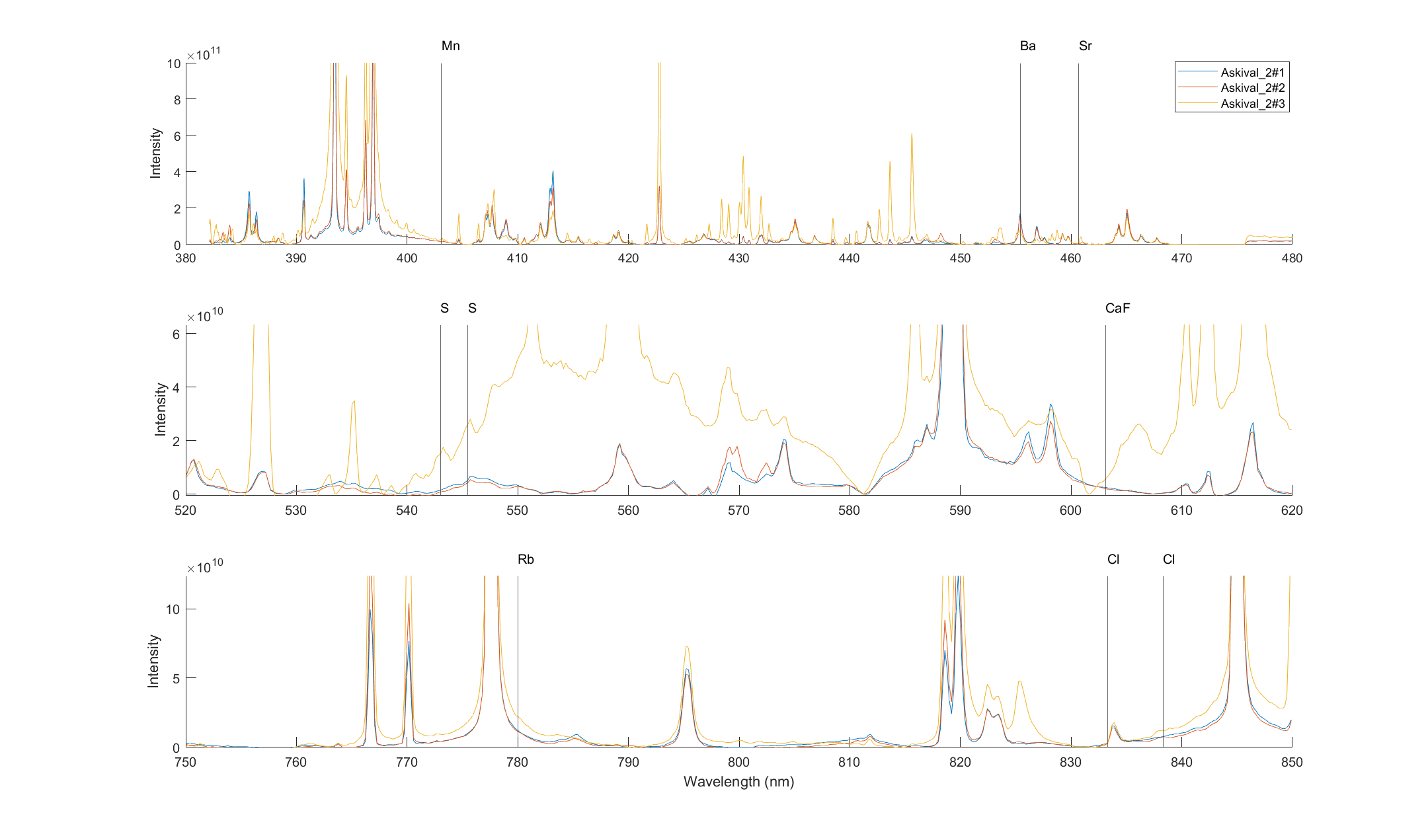


**Supplementary Figure 3: Askival_3 Raster Spectra**


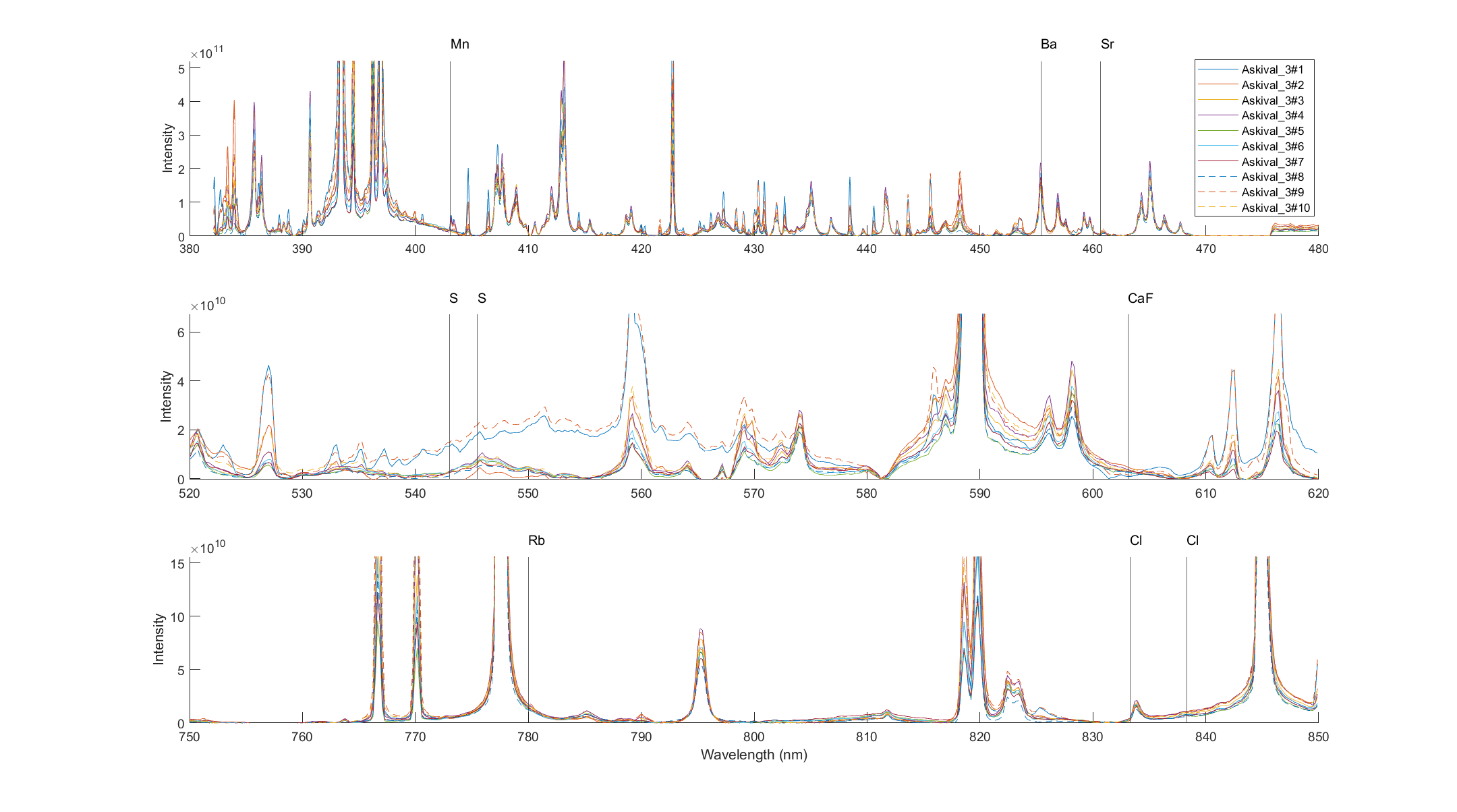

Supplement: Supplementary file 2 — Appendix S2. Askival ChemCam spectra. [file MAPS-58-41-s002.docx]
